# Assessing Inequities in COVID-19 Vaccine Roll-Out Strategy Programs: A Cross-Country Study Using a Machine Learning Approach

**DOI:** 10.3390/vaccines10020194

**Published:** 2022-01-26

**Authors:** Mehrdad Kazemi, Nicola Luigi Bragazzi, Jude Dzevela Kong

**Affiliations:** Africa-Canada Artificial Intelligence and Data Innovation Consortium (ACADIC), Department of Mathematics and Statistics, Faculty of Science, York University, Toronto, ON M3J 1P3, Canada; mehrdadk@my.yorku.ca (M.K.); bragazzi@yorku.ca (N.L.B.)

**Keywords:** COVID-19, pandemic, vaccine roll-out, cross-country analysis, machine learning, Random forest

## Abstract

After the start of the COVID-19 pandemic and its spread across the world, countries have adopted containment measures to stop its transmission, limit fatalities, and relieve hospitals from straining and overwhelming conditions imposed by the virus. Many countries implemented social distancing and lockdown strategies that negatively impacted their economies and the psychological wellbeing of their citizens, even though they contributed to saving lives. Recently approved and available, COVID-19 vaccines can provide a really viable and sustainable option for controlling the pandemic. However, their uptake represents a global challenge due to vaccine hesitancy and logistic–organizational hurdles that have made its distribution stagnant in several developed countries despite several appeals by the media, policy- and decision-makers, and community leaders. Vaccine distribution is also a concern in developing countries, where there is a scarcity of doses. The objective of the present study was to set up a metric to assess vaccination uptake and identify national socio-economic factors influencing this indicator. We conducted a cross-country study. We first estimated the vaccination uptake rate across countries by fitting a logistic model to reported daily case numbers. Using the uptake rate, we estimated the vaccine roll-out index. Next, we used Random Forest, an “off-the-shelf” machine learning algorithm, to study the association between vaccination uptake rate and socio-economic factors. We found that the mean vaccine roll-out index is 0.016 (standard deviation 0.016), with a range between 0.0001 (Haiti) and 0.0829 (Mongolia). The top four factors associated with the vaccine roll-out index are the median per capita income, human development index, percentage of individuals who have used the internet in the last three months, and health expenditure per capita. The still-ongoing COVID-19 pandemic has shed light on the disparity in vaccine adoption across low- and high-income countries, which represents a global public health challenge. We must pave the way for universal access to vaccines and other approved treatments, regardless of demographic structures and underlying health conditions. Income disparity remains, instead, an important cause of vaccine inequity, which restricts the functioning of the global vaccine allocation framework and, thus, the ending of the pandemic. Stronger mechanisms are needed to foster countries’ political willingness to promote vaccine and drug access equity in a globalized society where future pandemics and other global health crises can be anticipated.

## 1. Introduction

Since its initial outbreak in late December 2019, the still-ongoing “Coronavirus Disease 2019” (COVID-19) pandemic, caused by the infectious agent known as “Severe Acute Respiratory Syndrome-related Coronavirus type 2” (SARS-CoV-2), has been representing a true global public health challenge [1].

Due to the emerging nature of the pathogen, against which populations were largely immunologically naïve, and its highly contagious and quick-spreading nature, healthcare facilities have been overwhelmed by a high toll of infections [2]. To contain the virus and to counteract this novel strain, countries have implemented non-pharmaceutical interventions (NPIs), such as enhanced hygiene practices, social distancing, self-isolation, quarantine, and lockdown of entire territories [3].

Different from the early phases and waves when the entire world was completely unprepared to tackle the outbreak and NPIs were the only possible strategy to implement, as of today, several vaccines have been approved and are available. While NPIs, despite being effective in flattening the epidemic curve and curbing cases and deaths, are not sustainable in the long period, COVID-19 vaccines can provide a viable option and strategy for controlling the pandemic [4].

However, despite vaccines’ excellent effectiveness and safety profiles [5], mass immunization campaigns are successful only when the vaccine uptake rate is satisfactorily high, ensuring the achievement of herd immunity. This can, on the one hand, enable the control of the viral transmission dynamics, and, on the other hand, confer immune protection to those frail subjects, a significant portion of which, although being willing, are unable to vaccinate against COVID-19 because their status of immune deficiency or suppression does not enable them to build up sufficiently robust immunity levels [6].

Besides clinical reasons, other factors, such as lack of confidence towards science and vaccination, false beliefs regarding the severity of COVID-19 and/or the efficacy of vaccines, and structural and/or perceived barriers to immunization, can jeopardize the implementation of mass vaccination campaigns, thus posing serious health risks [6]. Indeed, the World Health Organization (WHO) defines vaccine acceptance as one of the major challenges to global public health [6].

Vaccine hesitancy is a complex, multi-factorial phenomenon, which results from the subtle, non-linear interplay among various parameters, ranging from socio-economic and educational variables to behavioral ones [7,8,9].

Specifically concerning COVID-19, this pandemic has been affecting more than 220 countries and territories. This has significantly impacted various healthcare sectors, including the chains of manufacturing and delivery of drugs and, in particular, vaccines’ supply, logistics, and distribution [10]. While many high-income countries have already started implementing immunization campaigns, securing for themselves more than half of the world’s available doses of COVID-19 vaccines, most developing countries, including African nations, are still waiting for vaccine stocks while preparing their vaccination campaigns [11].

Poor nations have to rely mainly on global collaborative co-financing vaccine procurement mechanisms, such as the “COVID-19 Vaccines Global Access” (COVAX) and the World Bank and the African Union’s COVID-19 “Africa Vaccine Acquisition Task Team” (AVATT) platforms, aimed at supporting equitable and sustainable access to COVID-19 vaccines [12].

To quantitatively assess inequities in vaccine allocation, distribution, and uptake at the global level, we conducted a cross-country study, employing machine learning techniques to assist and inform the modeling of non-linearity underlying the phenomenon of vaccine hesitancy. As such, this study can have important practical implications for global and public health workers, decision- and policymakers, and all relevant stakeholders involved in vaccine roll-out strategy programs.

## 2. Materials and Methods

### 2.1. Estimating the Vaccination Uptake Rate among Different Countries

**Figure 1:**
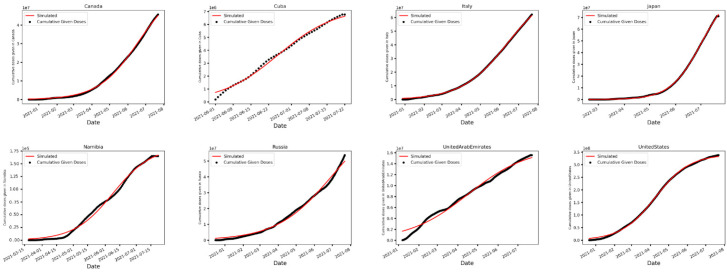
Dots represent cumulative given doses, and curves are fitted based on the logistic growth model. Countries are sorted alphabetically. Here, only some select countries are shown. The average of *R*^2^ across all countries is 0.99.

First, we computed the vaccination uptake rate across different countries. Since the growth of cumulative given doses is qualitatively similar to a logistic function that grows exponentially at first but slows as it proceeds reaching a plateau eventually (as shown in Figure 1), the logistic growth model was used to estimate the vaccination uptake rate. In the logistic model, the cumulative number of doses administered *c*(*t*) satisfies the following equation:

(1) $$c\left(t\right)=\frac{K}{1+a\cdot e^{-r\cdot t}}$$

where *K* is the total number of COVID-19 vaccine doses administered at the end of the vaccination campaign, *r* is the vaccination uptake rate, and $\frac{K}{1+a}$ = c(0) is the initial number of doses given. To estimate *r*, the least square fitting algorithm was employed through Python’s SciPy curvefit() function to fit the rate of change in cumulative cases of a logistic growth model to daily given doses based on the data from [13].

### 2.2. Vaccine Roll-Out Index (VRI)

Next, we defined the Vaccine Roll-out Index (*VRI*) for a country as follows:

(2) $$VRI=r\cdot \frac{d}{N}$$

where *r* is the vaccination uptake rate as defined in the previous paragraph, *d* is the total number of given doses, and *N* is the population. *VRI* was used as an index to compare the overall vaccination adoption among different countries, as it reflects both the speed and the extent of mass vaccination in a country. For instance, at the time of drafting the present paper, Haiti has the highest vaccination uptake rate (0.39) among all the countries compared. However, when it comes to the extent of vaccination campaign or *d*/*N*, it comes last (0.0004). This extreme example illustrates the importance of both speed and extent in shaping a successful vaccination campaign. For each country, *VRI* was calculated based on the estimated *r* values and publicly available data [14] for *d*/*N*.

### 2.3. Covariates

Next, through a comprehensive literature review and assessment [15,16], critical thinking, and consultations with experts, 36 covariates from ten different categories (namely, (i) demographic, (ii) disease, (iii) economic, (iv) environmental/habitat, (v) health, (vi) education, (vii) technology, (viii) social, (ix) health/social, and (x) composite index—economic/social/health/education) were chosen as predictors for *VRI* [13,14,17,18,19,20,21,22,23,24,25,26,27,28,29,30,31,32,33,34,35,36]. To the best of our knowledge, this number of covariates is unprecedented in the existing scholarly literature since many similar studies have used few covariates. Moreover, these studies employed linear regression. However, due to the use of the Random Forest (RF) algorithm, we were able to include non-linear covariates as well since, as previously mentioned, vaccine hesitancy is a complex, multi-factorial, non-linear phenomenon.

We built a data set with 142 countries accounting for 95% of the world’s population. We collected the most recent available data on the chosen covariates from publicly available databases after checking for their accuracy, reliability, and completeness. We selected diverse, specific covariates that are comparable across countries. For instance, nurses per capita as a variable was favored over doctors per capita since healthcare systems can vary among countries, and nurses are the primary care provider in many countries. Additionally, for a better comparison, we divided absolute numbers by total population to obtain per capita numbers. To deal with missing data, for each covariate, we used a Classification and Regression Trees (CART) algorithm to estimate missing data based on the other covariates. Below is the table of all the covariates used in this study with their explanations and sources (Table 1).

### 2.4. Random Forest-Based Regression Analysis of the Association between Covariates and VRI

Random Forest (RF), an “off-the-shelf” machine learning algorithm, was used to determine associations between predictors and *VRI*. Random forest is a collection of decision trees where each tree depends on the value of an independently sampled vector chosen randomly (Breiman, 2001) [37]. For regression, RF implements a combination of de-correlated decision trees and declares the final output as the average of all predictions made by trees. As opposed to the prevalent literature, we decided to use RF for modeling, as it has many advantages that make it a perfect fit for this type of data analysis. Such advantages include [38]:

- Can capture non-linear relationships between features and the target variable
- Less sensitive to outliers
- No need to prune the decision trees (overcoming the issue of overfitting)
- The importance of each covariate can be numerically measured
- Can handle continuous, categorical, and binary data

**Figure 2:**
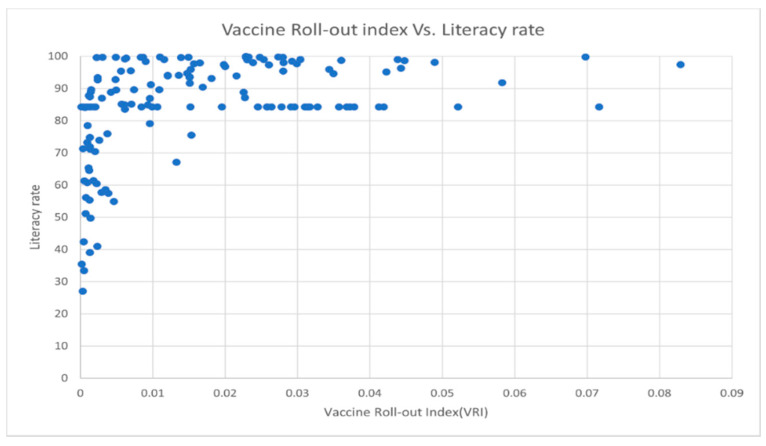
Vaccine Roll-out Index (*VRI*) versus Literacy rate (Adult literacy rate is the percentage of people aged 15 years and above who can both read and write with a clear understanding of a short simple statement about their everyday life).

Among all these advantages, the ability to capture non-linear relationships between features and the target variable is crucially important for this study. Non-linear relationships between variables are a common feature of many datasets. For instance, below is the plot of literacy rate versus *VRI*, which clearly does not show a linear pattern (Figure 2).

All the other scatterplots are shown in Figure S1.

We used the “RandomForestRegressor” module in the Python Scikit-learn library to build 500 decision trees where, for each tree, the square roots of the total number of covariates were chosen randomly to make splits [39]. In addition, the maximum depth of each tree was obtained by a 10-fold cross-validation. Since we were only interested in finding associations between covariates and the target variable, we used all the data as the training data. To assess the contribution of each covariate to the model fitting, we implemented [37]’s permutation-based measures. This method considers a feature to be important if shuffling its values increases the model error and unimportant if it does not change the model error too much.

### 2.5. Evaluation Metrics

In terms of evaluation, we used the mean squared error (*MSE*) and the coefficient of determination *R*^2^. *MSE* measures how close a prediction is to the observed value and is given by the formula:

(3) $$MSE=\frac{1}{n}\overset{n}{\underset{i=1}{\sum }}{\left(y_{i}-\hat{y_{l}}\right)}^{2}$$

where n is the sample size, $y_{i\;}$ is the observed value and $\hat{y_{l}}$ is the predicted one; $R^{2}$ is given by the formula:

(4) $$R^{2}=1-\frac{\sum_{i=1}^{n}{\left(y_{i}-\hat{y_{l}}\right)}^{2}}{\sum_{i=1}^{n}{\left(y_{i}-\bar{y_{n}}\right)}^{2}}$$

where $\bar{y_{n}}$ is the average of the observed values. $R^{2}$ represents the proportion of the dependent variable’s variance that is explained by independent variables in the model. For our model, which was trained using all the data only to find associations, MSE = 0.00002 and $R^{2}$ = 0.92, which proves the validity of our approach.

## 3. Results

### 3.1. Estimation of COVID-19 Vaccination Uptake Rate among Countries

Figure 1 and Table S1 show vaccination uptake curves fitted to observed time series of daily given doses across studied countries. In particular, Table S1 provides the estimated vaccination uptake rate across studied countries. For 142 countries considered, the average of *R*^2^ was 0.99 and vaccination uptake rate was highest in Haiti, Algeria, and Madagascar with 0.395, 0.239, and 0.173, respectively, and lowest in Turkey, Indonesia, and Eswatini with 0.0144, 0.0206, and 0.0208, respectively. Overall, the average vaccination uptake rate was found to be 0.046 with a standard deviation of 0.042. Moldova (0.04635), Vietnam (0.0464), and Georgia (0.045) were the closest to the mean.

### 3.2. Vaccine Roll-Out Index (VRI) among Countries

**Figure 3:**
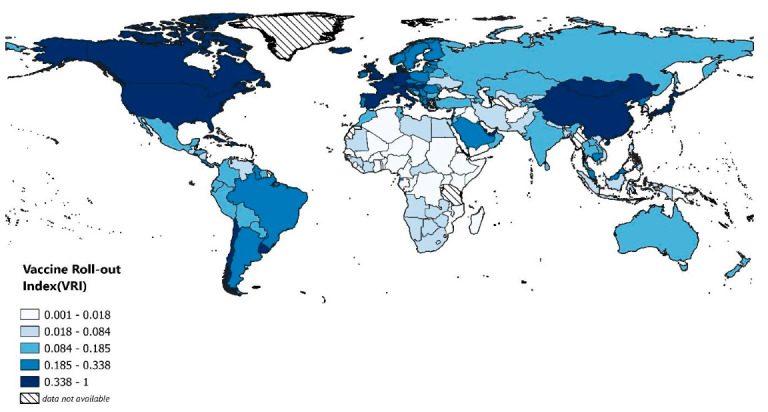
Heatmap of studied countries. They are colored based on their Vaccine Roll-out Index (*VRI*) value. All *VRI* values were multiplied by a constant to be in the range of 0 to 1. Five intervals were selected such that the number of countries in each one was the same.

Figure 3 and Table S2 summarize the value of *VRI* for countries studied. For 142 countries considered, *VRI* was highest in Mongolia (0.083), Israel (0.072), and Cuba (0.070) and lowest in Haiti (0.00014), Chad (0.00021), and South Sudan (0.00034). The mean *VRI* was 0.016 with a standard deviation of 0.016. Romania, Argentina, and Comoros were the closest to the mean with 0.0157, 0.0165, and 0.0153, respectively. Although it is counterintuitive that a developing country is leading, Mongolia has reportedly emerged as a positive outlier [40,41,42]. Due to its unique geopolitical situation, the country has been able to receive COVID-19 vaccine doses from its neighbors China and Russia [40,41,42]. Moreover, as a developing country, Mongolia has received doses from the COVAX as well [42]. Cuba is another outlier but in a different way. Unlike Mongolia, which relies on its neighbors, Cuba has a long successful history of vaccine development. Starting in the 1980s, Cuba’s state-owned, -funded, and -operated biotech sector was supported by the government, which provided at least a billion dollars. As of today, the country owns one of the world’s leading biotech industries, with more than 30 manufacturers and research institutes, which produce eight of the 11 vaccines needed for the country’s national immunization program. That being said, all of Cuba’s COVID-19 vaccines are subunit protein vaccines and are made by fermentation in mammalian cells, a process already used by the country for monoclonal antibodies. In addition, these vaccines are rather cheap to produce and do not require extreme refrigeration, which makes them easy to produce at a large scale.

### 3.3. Association between Predictors and VRI

**Figure 4:**
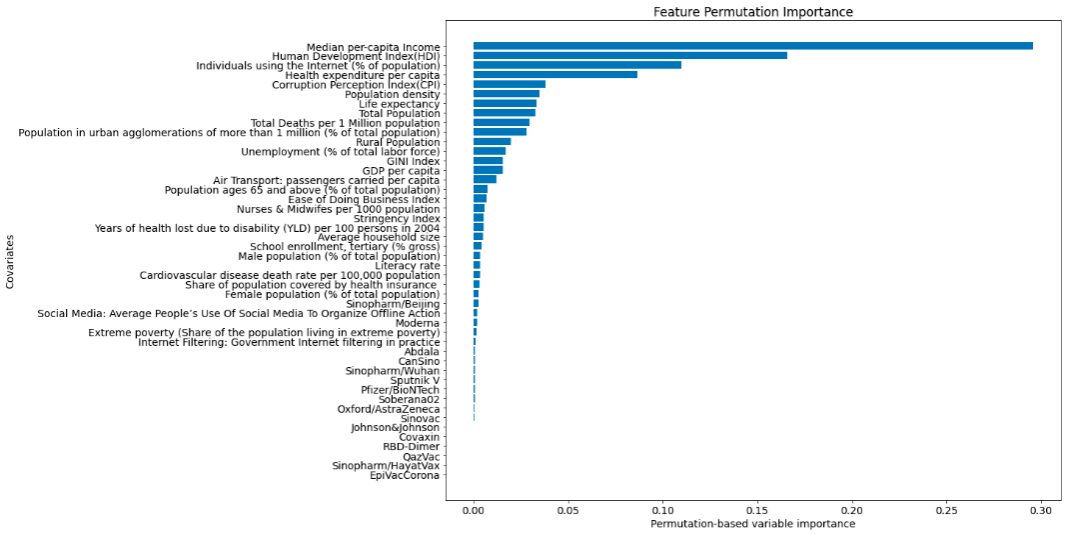
Covariates listed according to their Permutation Feature Importance. Four top features are: (i) median per capita income; (ii) human development index; (iii) the percentage of individuals who have used the internet in the last three months (latest data available) via a computer, mobile phone, personal digital assistant, games machine, digital TV, etc.; and (iv) health expenditure per capita. Additionally, the results illustrate that other covariates, particularly the type of vaccine or the Gini index, do not play a key role.

Figure 4 indicates that (i) median per capita income; (ii) human development index; (iii) percentage of individuals who have used the internet in the last three months (latest data available) via a computer, mobile phone, personal digital assistant, games machine, digital TV, etc.; and (iv) health expenditure per capita are the four most important covariates associated with the Vaccine Roll-out Index (*VRI*).

## 4. Discussion

The present study has shown that (i) median per capita income; (ii) human development index; (iii) the percentage of individuals who have used the internet in the last three months via a computer, mobile phone, personal digital assistant, games machine, digital TV, etc.; and (iv) health expenditure per capita are the four most important covariates associated with the Vaccine Roll-out Index (*VRI*).

These findings are in line with those of previous studies [43,44]. More specifically, Duan et al. [43] carried out a cross-sectional ecological study and analyzed the association between country income level and COVID-19 vaccination coverage rates in 138 countries in terms of the mediating role of vaccination policies. The authors devised a single-mediator model based on structural equation modeling. The authors found that, with respect to high-income countries, upper-middle-, lower-middle-, and low-income countries reported lower vaccination coverage rates. Immunization policies were found to mediate from 14.6% to 15.6% of coverage in upper-middle and lower-middle countries, respectively, whereas this effect was not statistically significant in low-income countries. Conclusions were similar when accounting for different country-related demographic and health parameters. Roghani and Panahi [44] quantitatively assessed the association of COVID-19 vaccine allocation and two major macro-socioeconomics measures, namely, HDI and Gross domestic product (GDP), in 25 countries. The authors found a positive, statistically significant association between GDP per capita and COVID-19 vaccine distribution, while no link could be detected for HDI.

With respect to these two studies, our investigation is much broader and takes into account more countries and more covariates, utilizing a sophisticated machine learning approach that enables us to model the non-linearity underlying the phenomenon of vaccine hesitancy. However, despite some methodological differences, all this suggests that high-income countries, widely known as developed countries [45], are more likely to have higher COVID-19 vaccine adoption rates among all countries.

This is precisely consistent with the way COVID-19 vaccination adoption has been unfolding worldwide. By late April, more than 81% of the doses had been administered to people residing in high- and upper-middle-income countries, with only 0.3% being received by people in low-income countries [46]. In fact, economically developed countries have secured enough doses to vaccinate 245% of their adult populations [47]. As a result, low- and lower-middle-income countries can only cover around one-third of their citizens with purchased doses [47]. Hence, low-income countries are not expected to reach vaccination herd immunity until 2023, if at all [48]. Therefore, low-income nations are most likely to continue suffering from COVID-19 for a longer span of time compared to high-income countries [49].

COVID-19 vaccine allocation, distribution, and deployment are significantly uneven with around 95% of the total doses being administered to only 20% of the global population [50]. The observation that in low-income countries, clinically vulnerable and frail individuals are dying from COVID-19, yet simultaneously, already fully vaccinated people are lining up to get their third dose in wealthy countries, has been labeled as “a catastrophic moral failure” by the Director-General of the WHO, who has called for a moratorium in the administration of booster shots to help developing countries, which are struggling to vaccinate against COVID-19.

Ensuring that low-income countries have sustainable access to COVID-19 vaccines should be regarded as a global responsibility and onus [51]. It must be noted that the challenge that developing countries are facing is not limited to vaccination but also in procuring other supplies, such as chemical reagents necessary for testing for COVID-19 as well as approved drugs that were thought to be helpful in treating COVID-19 or, at least, mitigating against the severity of its symptoms [49]. In addition, even if a low-income country succeeded to purchase vaccines, they would face challenges to storing and even administering the doses [49]. In fact, according to a recently published report from the World Bank, “vaccine preparedness” is a challenge in many low- and middle-income countries [52]. One of the main reasons why high-income countries can hoard vaccines through firsthand access is due to their huge investments in vaccine development [49]. This would have been too expensive and unaffordable for many developing countries.

Based on a technical report [53], about £2.5 million for Clinical Trial Phase I, £20 million for Clinical Trial Phase II, £65–250 million for Clinical Trial Phase III, and £20 million for Phase IV are needed. Adding it up, this number could reach as high as £292.5 million. The COVID-19 pandemic is a novel and unprecedented situation, but the inequality in access to vaccines as well as other healthcare provisions has been a common theme throughout many past pandemics and outbreaks.

The COVID-19 pandemic will continue to be a threat until every citizen is covered and fully immunized in both rich and poor nations [49]. Scientists have warned that unless eradicated, the pandemic will evolve into a pan-endemic infection with a probable resurgence as late as 2024 [54,55]. Biologically, along with this disparity in vaccine adoption across different countries, new vaccine-resistant variants of concern (VOCs) will continue to emerge and sequentially threaten high-income countries as well [56,57,58].

Therefore, completely ending the COVID-19 pandemic and recovering global economy call for global access to vaccines and other effective drugs [49]. Some approaches to reducing the gap between low- and high-income countries’ vaccine adoption include, but are not limited to, bilateral and multilateral donations and charity, scaling-up of vaccine production, and temporary waivers of intellectual property [49]. To vaccinate priority groups in all countries around the world, at least 1.3 billion doses are needed for 92 low- and middle-income country members of the COVAX platform initiative [58]. Researchers and scholars from the Kaiser Family Foundation (KFF) have stated that, without redistribution and re-allocation of doses already purchased by economically developed countries and/or enhanced support for manufacturing or production of further additional doses, globally, more than four out of ten (41%) adults will not be able to be immunized, even after allocating all COVAX doses to low- and middle-income countries [47]. Lastly, since in order to end inequities in vaccine uptake, the root causes of global health inequities must be the target to change [58], the findings of this study may greatly help policy- and decision-makers and stakeholders on their mission to decrease the inequality in vaccine adoption across different countries worldwide. Our results reaffirm the current inequality in global health, deeply rooted in the unbalanced universal distribution of wealth.

Additionally, interestingly, the results point out that overall, the type of vaccine does not play a key role in vaccination adoption for many countries. A plausible interpretation of this could be that most people simply do not have a choice. In fact, as only 29.1% of the world has received at least one dose of COVID-19 vaccines, they would be lucky if they got one.

Intriguingly, the Gini index, which is an indicator measuring the degree of inequality in the income/wealth distribution and is computed as the proportion of the total income/wealth of the population cumulatively earned by the bottom percentage (*x*) of the population, was not found to be associated with the *VRI*. Even though surprising, this finding is in line with other studies, including the investigation by Sobral and colleagues [59].

## 5. Strengths and Limitations of the Study

One limitation of this study is that, due to limited available data on the panel of vaccines administered by each country, we did not consider the effectiveness of vaccines used by countries. In addition, for all countries, we used the number of given doses divided by population in the formula of *VRI*. This assumes that all the vaccines used by a country need the same number of doses to reach their full effectiveness, which is not the case with the Johnson & Johnson/Janssen vaccine, which is a single-dose vaccine product.

Another limitation is that inequalities in vaccine uptake could reflect the presence of political, social, and religious leaders, advising against vaccination, in countries like Brazil, the US, and Romania, among others [60,61,62]. The existing scholarly literature has shown, indeed, that COVID-19 immunization rates have become politically highly polarized, with a significant percentage of US Republicans remaining vaccine-hesitant for several months. Cues and endorsements by party elites can affect COVID-19 vaccination intentions and attitudes [60]. The phenomenon of political influences is temporally variable and unstable and, as such, difficult to model and incorporate in the present study.

A further shortcoming is given by the very study design implemented in this paper (cross-country analysis); as such, the investigation suffers from what is called “ecological fallacy” or “ecological bias”. On the other hand, the explorative nature of the paper has enabled us to discover new patterns and associations/relationships among variables.

Finally, we have to acknowledge that there is a significantly high amount of heterogeneity across the countries investigated in the present papers in terms of widely different political systems, regionality of governance (federal versus central governance), the nature of resource control (socialist versus capitalist), and the level of transparency and corruption. Moreover, the list of covariates is far from being exhaustive, and, in future studies, further variables could be incorporated, such as the “Consumer Price Index” (CPI) or the “Global Competitiveness Index” (GCI), among others.

The novelty and strength of our work reside in the use of RF to find associations, which enabled us to include 36 covariates with both linear and non-linear relationships to the target variable. To the best of our knowledge, this method has never been used in previous studies, making the insights from this research more valuable.

## 6. Conclusions

The still ongoing COVID-19 pandemic has shed light on the chronic inequality in global health systems. The disparity in vaccine adoption across low- and high-income countries is a challenge to the achievement of many global goals, such as the “Sustainable Development Goal” (SDG) 3, concerning “Good Health and Well-being”, which is one of the 17 SDGs established by the United Nations in 2015 [48]. Our investigation confirms that the median per capita income is the main contributor to the inequality in vaccine adoption across different countries.

As a lesson learned from this global crisis, we must pave the way for universal access to vaccines and other approved treatments as mentioned in SDG 3, regardless of demographic structures and underlying health conditions. Income disparity remains, instead, an important cause of vaccine inequity, which restricts the functioning of the global vaccine allocation framework and, thus, the ending of the pandemic. Stronger mechanisms are needed to foster countries’ political willingness to promote vaccine and drug access equity in a globalized society, where future pandemics and other global health rises can be anticipated.

## Figures and Tables

**Table 1:** List of all the covariates used in this study with their explanations and sources.

Category	Covariate	Source
Demographic	Youth: Population aged 20–35 years (% of the total population)	[17]
	Total Pop: Total Population	[17]
	Population density	[17]
	Median age	[18]
	Aged 65 years and older	[17]
	Rural population	[27]
	Gender ratio	[28]
	Average household size (number of members)	[18]
Disease	Mort Resp: Mortality rate from lower respiratory infections (per 100,000 people)	[19]
	Mortality rate from infectious and parasitic diseases (per 100,000 people)	[19]
Economic	GINI: GINI index	[20]
	Ease of doing business index 2019 (1 = most business-friendly regulation)	[21]
	GDP per capita	[17]
	Extreme poverty (share of the population living in extreme poverty, most recent year available since 2010)	[17]
	Median per-capita Income	[32]
	Unemployment, total (% of the total labor force)	[33]
Habitat	Population in urban agglomerations of more than 1 million (% of the total population)	[17]
	Urban population (% of the total population)	[17]
Health	GHS: Global Health Security detection index	[22]
	Nurses: Nurses and midwives (per 1000 people)	[17]
	Stringency index	[25]
	Total deaths attributed to COVID-19 per 1,000,000 people	[26]
	Type of vaccine	[13]
	Health expenditure per capita, PPP	[29]
	Share of the population covered by health insurance	[30]
	Cardiovascular disease death rate (per 100,000 people)	[31]
	Years of health lost due to disability (YLD)	[35]
Education	Literacy rate (percentage of people aged 15 years and above)	[17]
	School enrollment, tertiary (% gross)	[34]
Technology	Individuals using the Internet (% of the population)	[17]
Social	Social Media: Average People’s Use of Social Media To Organize Offline Action (4 = high)	[23]
	Internet Filtering: Government Internet filtering in practice (4 = low)	[23]
	Air Transport: passengers carried per capita	[17]
	Corruption Perceptions Index (CPI)	[36]
Health-Social	Life expectancy (Life expectancy at birth in 2019)	[24]
Composite index (Economic-Social-Health-Education)	Human development index	[14]

## Data Availability

All data are available within the manuscript and the supporting information, which is available as supplementary material.

## Supplementary Materials

The following supporting information can be downloaded at: https://www.mdpi.com/article/10.3390/vaccines10020194/s1, Figure S1: Scatterplots showing the relationships between the Vaccine Roll-Out Index (*VRI*) and the covariates under study; Table S1: Estimated vaccination uptake rates across studied countries; Table S2: Vaccine Roll-Out Index (*VRI*) values for the countries studied in the present investigation.
