# Supplementary material for: Assessing Inequities in COVID-19 Vaccine Roll-Out Strategy Programs: A Cross-Country Study Using a Machine Learning Approach
Source: Vaccines (Basel). 2022 Jan 26;10(2):194. doi: 10.3390/vaccines10020194 (PMC8879459; doi:10.3390/vaccines10020194)
Supplement: Supplementary file 1 [file vaccines-10-00194-s001.zip › vaccines-1468024-supplementary.pdf]

Figure S1. scatterplots showing the relationships between the Vaccine Roll-Out Index (VRI) and the covariates under study.

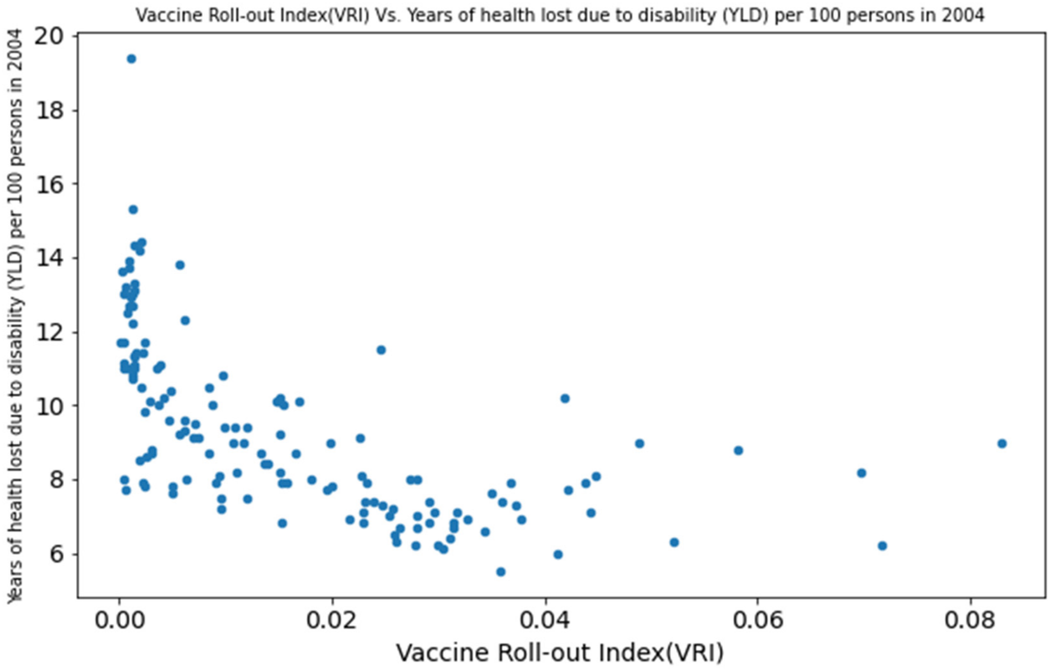

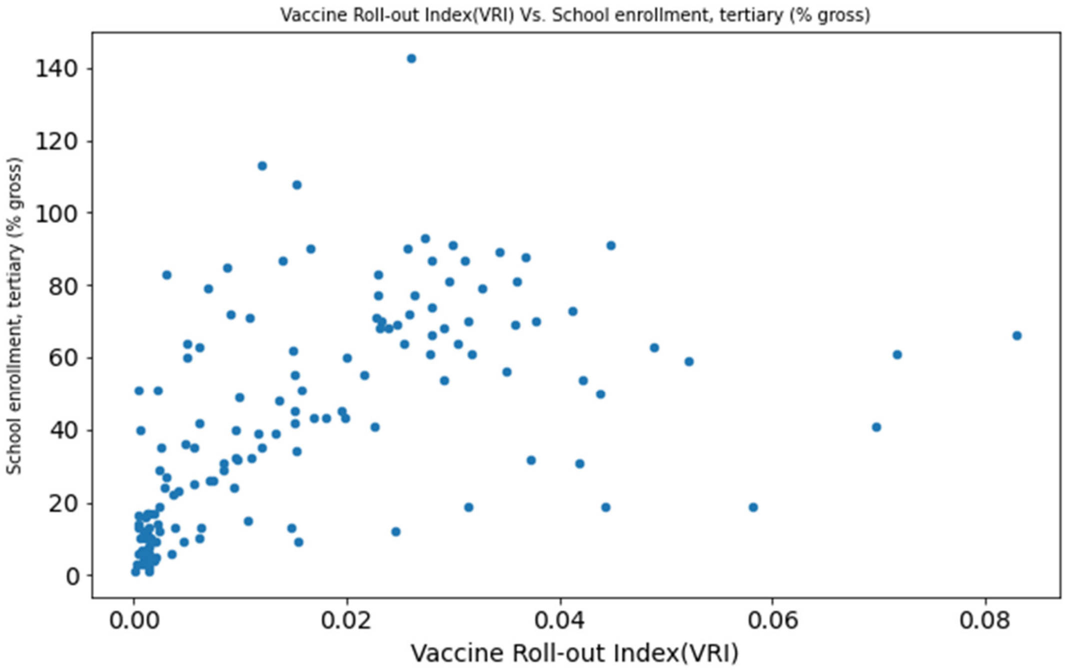

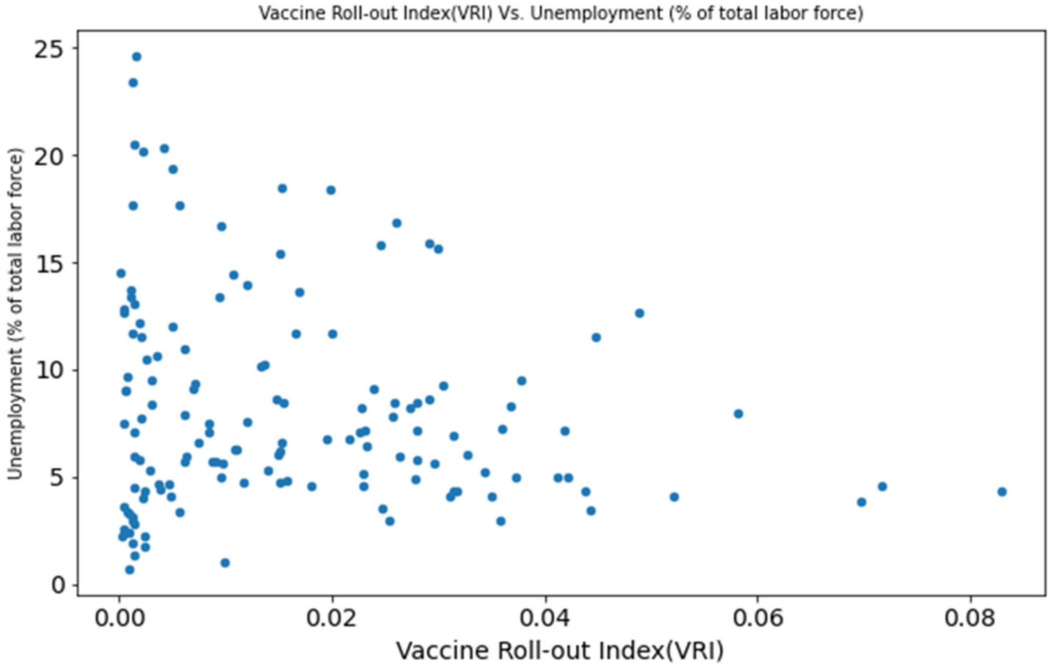
Vaccine Roll-out Index(VRI) Vs. Unemployment (% of total labor force)
Unemployment (% of total labor force)
0
5
10
15
20
25
0.00
0.02
0.04
0.06
0.08
Vaccine Roll-out Index(VRI)

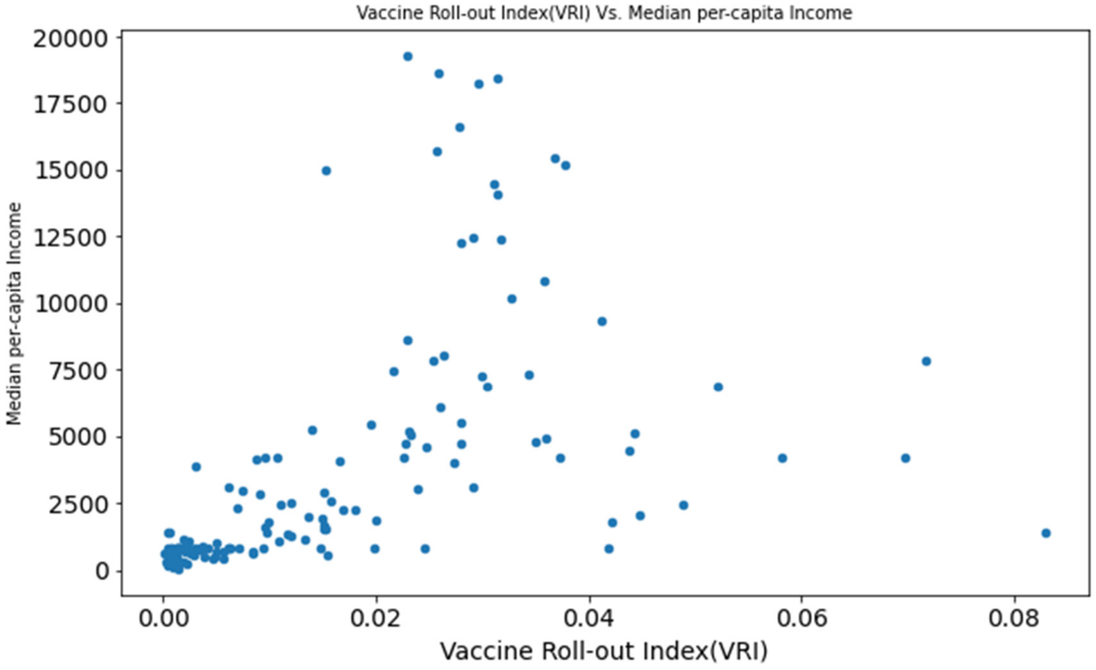
Vaccine Roll-out Index(VRI) Vs. Median per-capita Income
Median per-capita Income
0
2500
5000
7500
10000
12500
15000
17500
20000
0.00
0.02
0.04
0.06
0.08
Vaccine Roll-out Index(VRI)

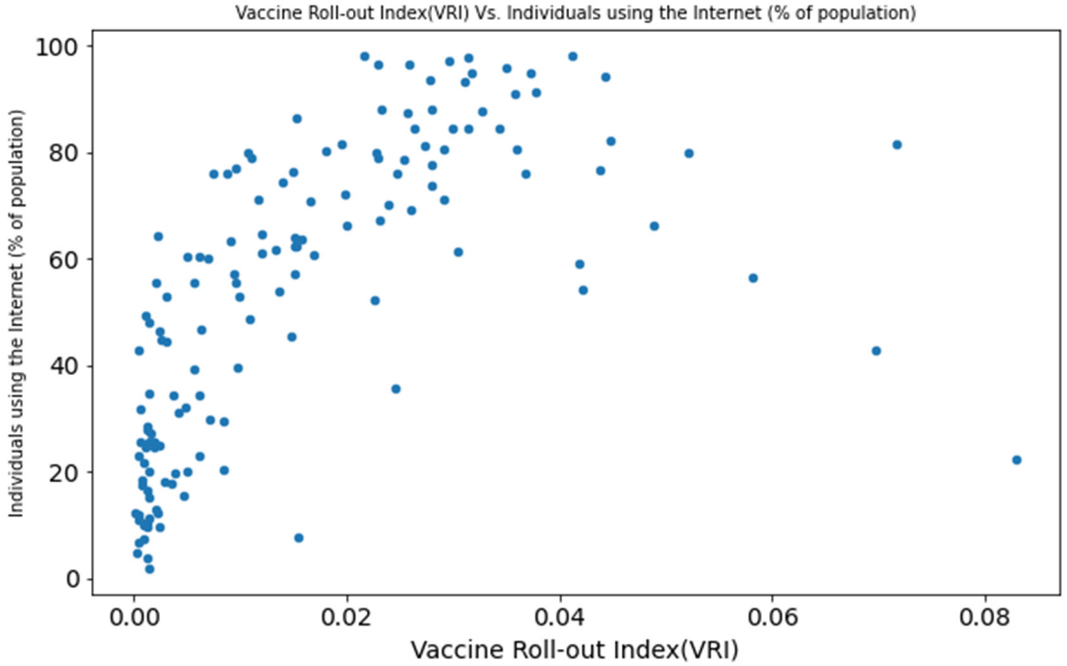
Vaccine Roll-out Index(VRI) Vs. Individuals using the Internet (% of population)
Individuals using the Internet (% of population)
Vaccine Roll-out Index(VRI)
0
20
40
60
80
100
0.00
0.02
0.04
0.06
0.08

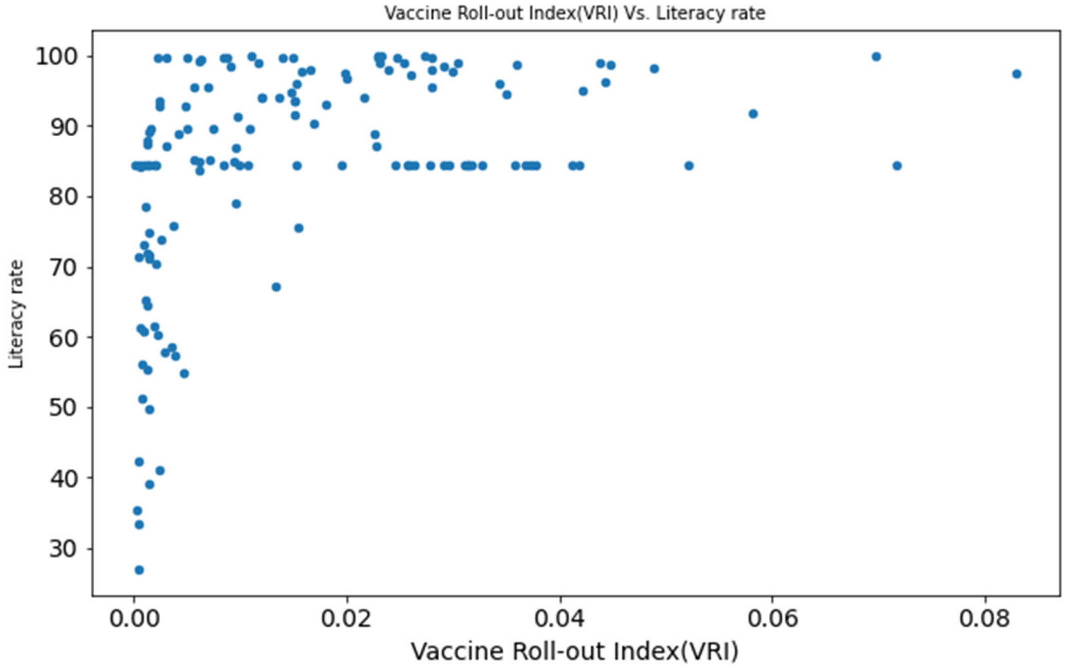
Vaccine Roll-out Index(VRI) Vs. Literacy rate
Literacy rate
Vaccine Roll-out Index(VRI)
30
40
50
60
70
80
90
100
0.00
0.02
0.04
0.06
0.08

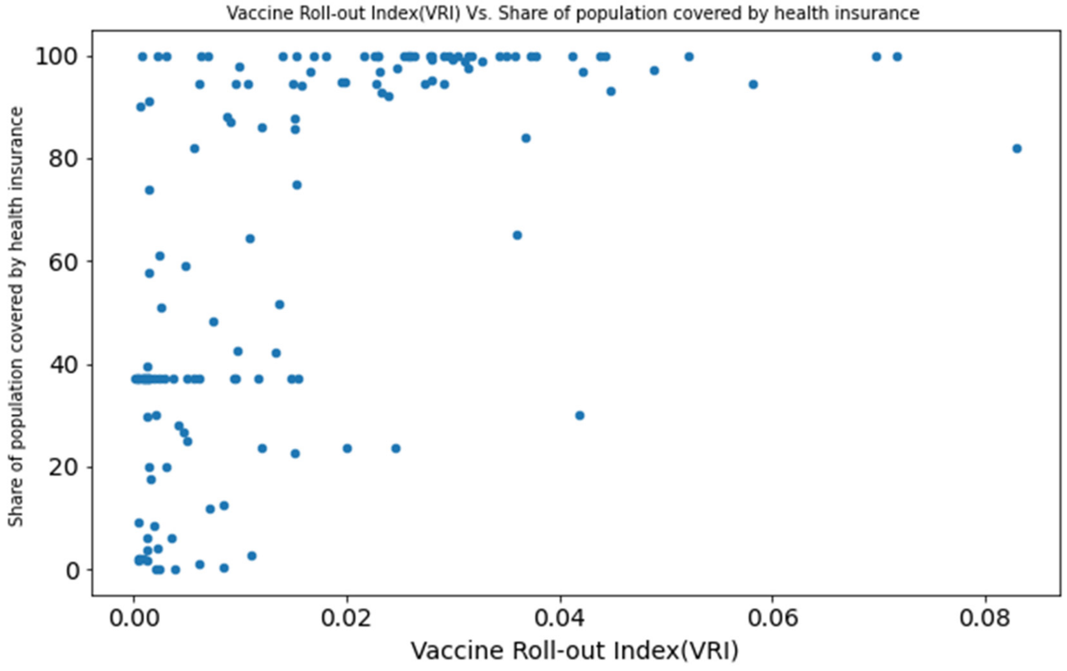
Vaccine Roll-out Index(VRI) Vs. Share of population covered by health insurance
Share of population covered by health insurance
Vaccine Roll-out Index(VRI)
0
20
40
60
80
100
0.00
0.02
0.04
0.06
0.08

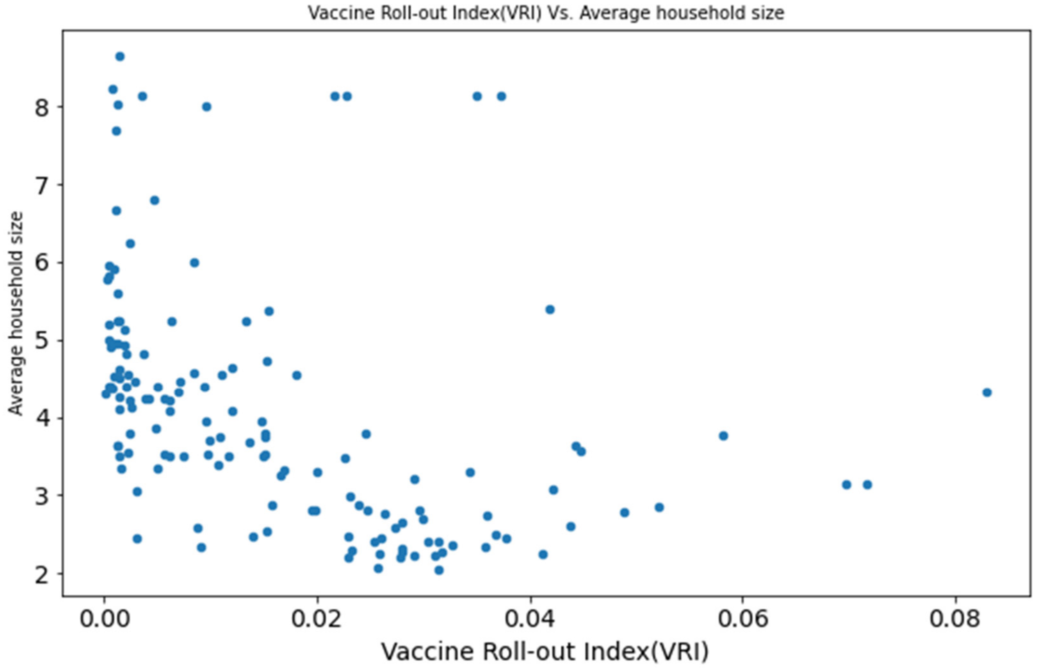
Vaccine Roll-out Index(VRI) Vs. Average household size
Average household size
Vaccine Roll-out Index(VRI)
2
3
4
5
6
7
8
0.00
0.02
0.04
0.06
0.08

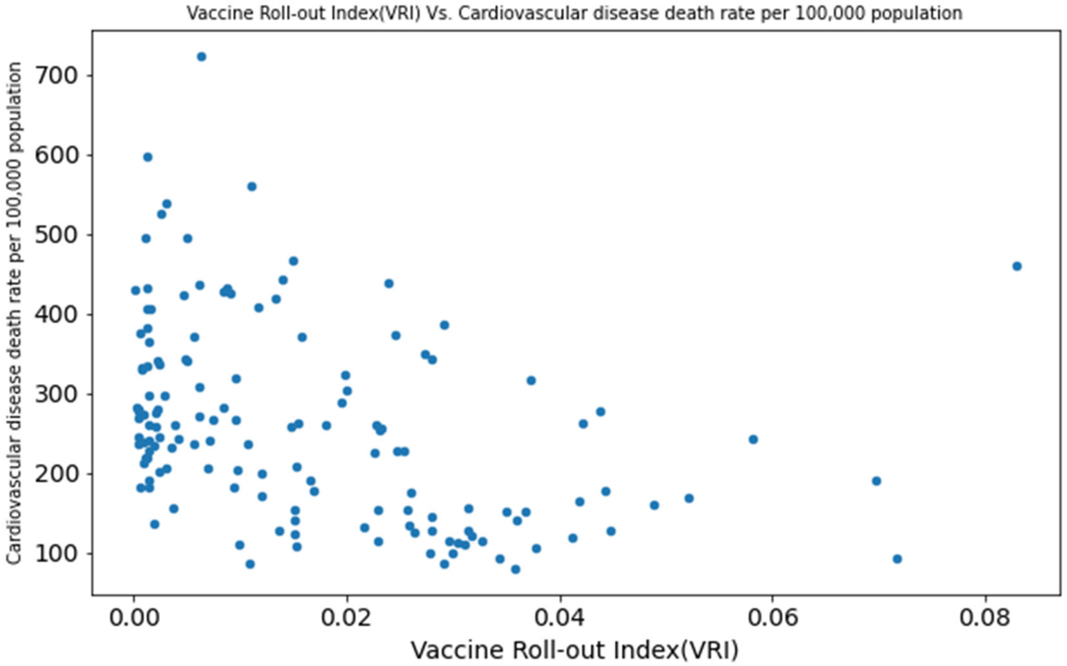
Vaccine Roll-out Index(VRI) Vs. Cardiovascular disease death rate per 100,000 population
Cardiovascular disease death rate per 100,000 population
Vaccine Roll-out Index(VRI)
100
200
300
400
500
600
700
0.00
0.02
0.04
0.06
0.08

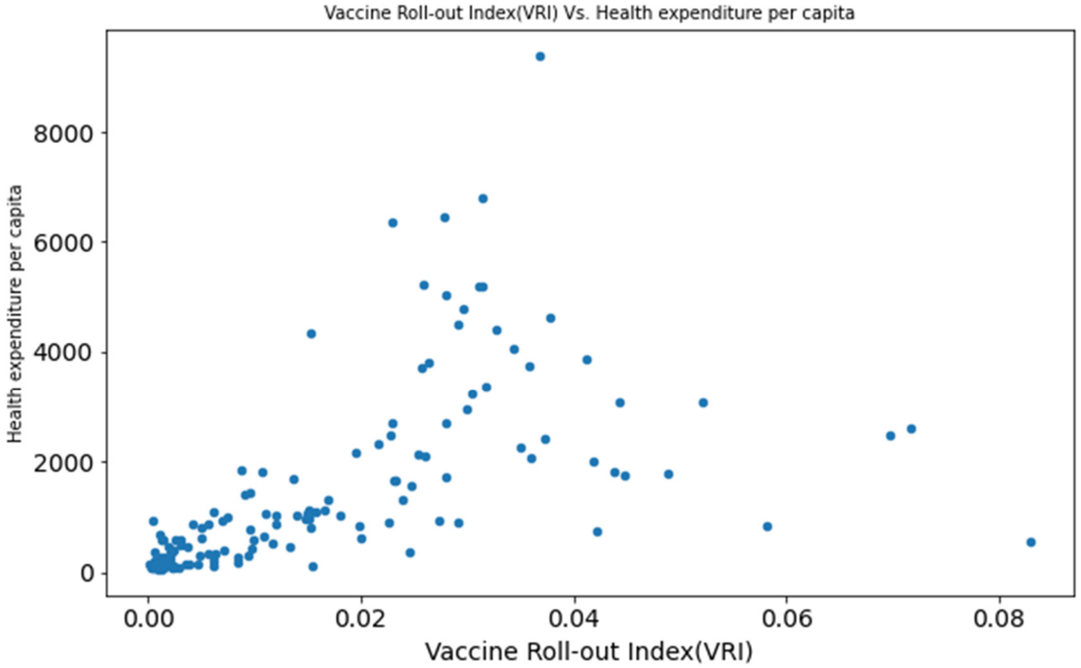
Vaccine Roll-out Index(VRI) Vs. Health expenditure per capita
Health expenditure per capita
Vaccine Roll-out Index(VRI)
0
2000
4000
6000
8000
0.00
0.02
0.04
0.06
0.08

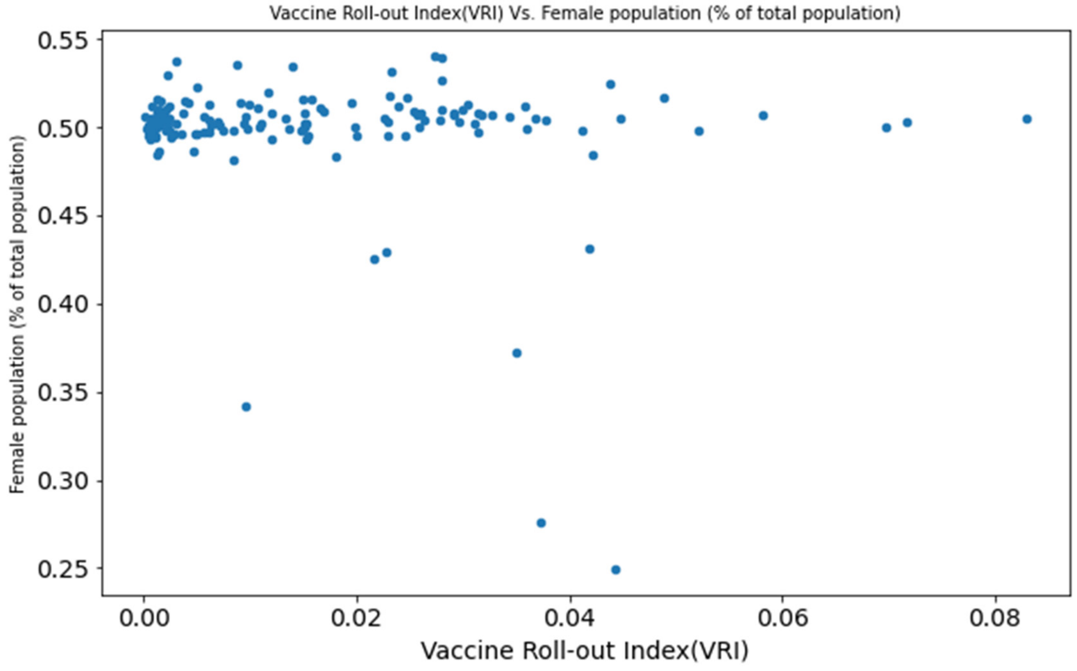
Vaccine Roll-out Index(VRI) Vs. Female population (% of total population)
Female population (% of total population)
0.55
0.50
0.45
0.40
0.35
0.30
0.25
0.00
0.02
0.04
0.06
0.08
Vaccine Roll-out Index(VRI)

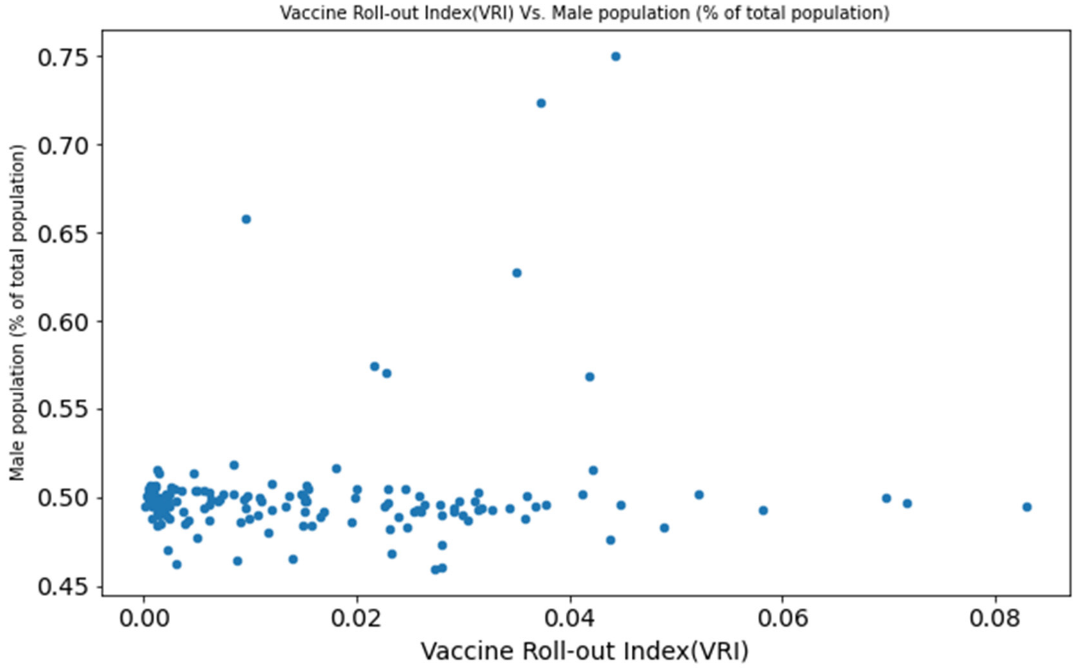
Vaccine Roll-out Index(VRI) Vs. Male population (% of total population)
Male population (% of total population)
0.75
0.70
0.65
0.60
0.55
0.50
0.45
0.00
0.02
0.04
0.06
0.08
Vaccine Roll-out Index(VRI)

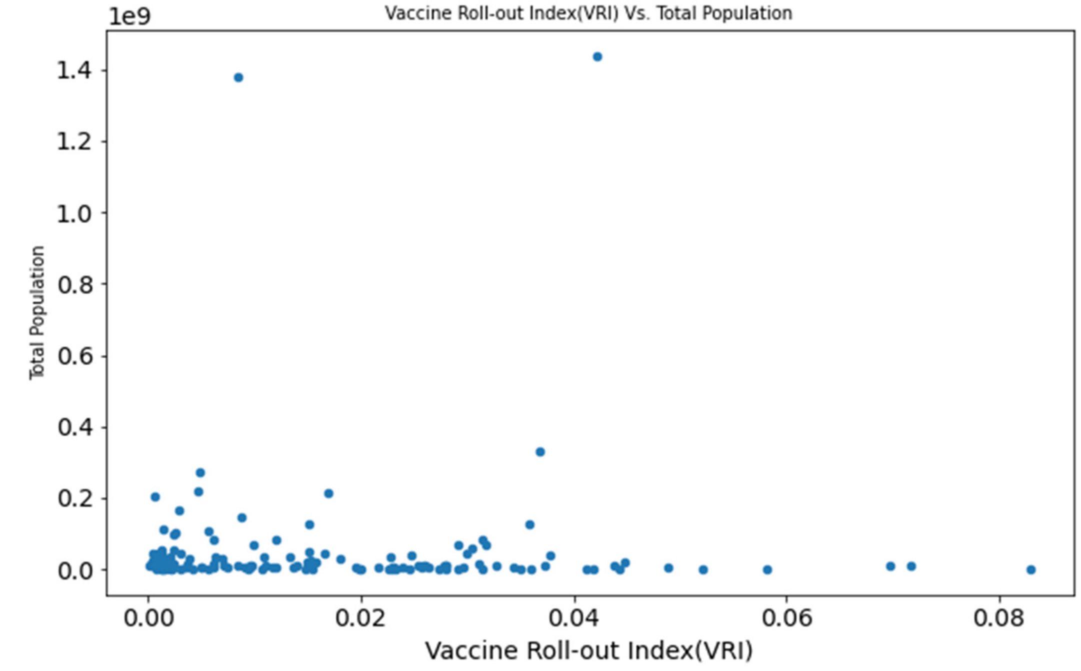

Vaccine Roll-out Index(VRI) Vs. Total Population
1e9
1.4
1.2
1.0
0.8
0.6
0.4
0.2
0.0
Total Population
0.00
0.02
0.04
0.06
0.08
Vaccine Roll-out Index(VRI)

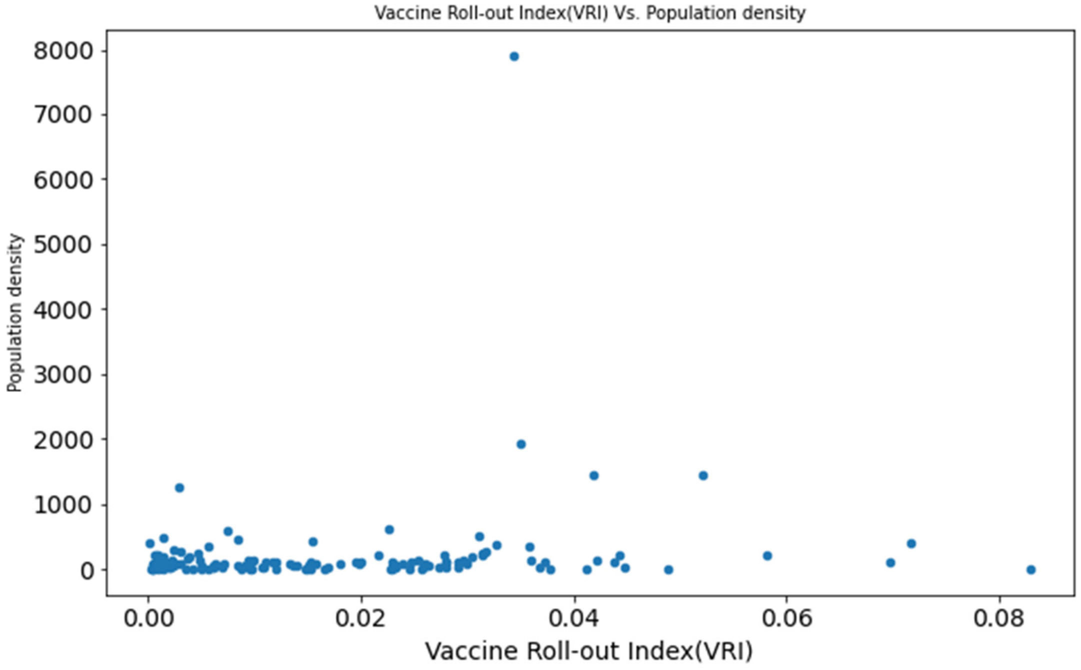

Vaccine Roll-out Index(VRI) Vs. Population density
8000
7000
6000
5000
4000
3000
2000
1000
0
Population density
0.00
0.02
0.04
0.06
0.08
Vaccine Roll-out Index(VRI)

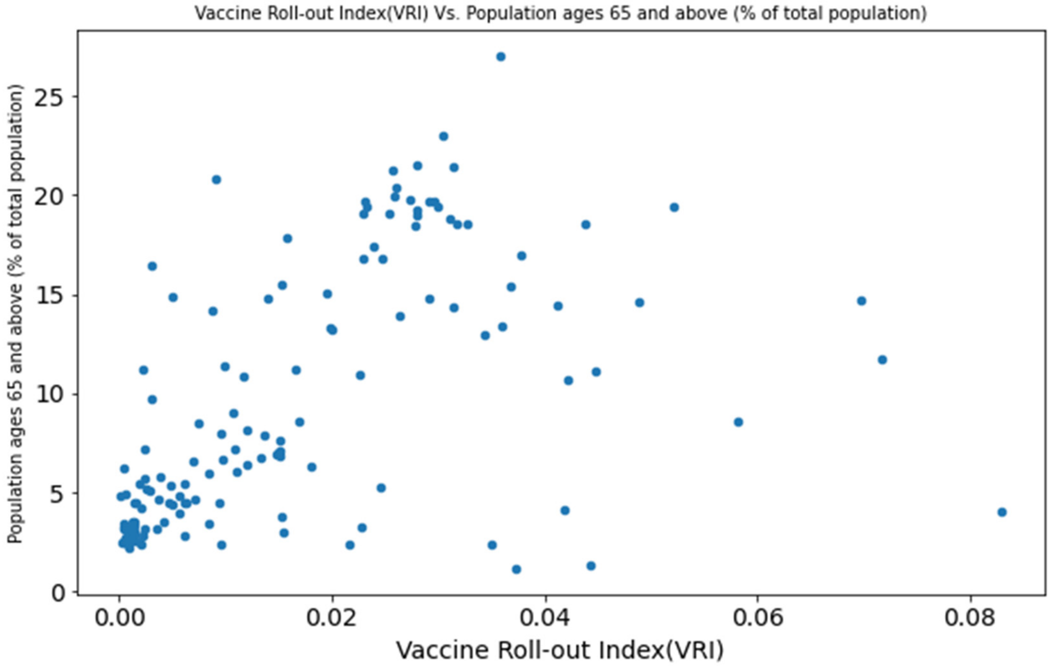
Vaccine Roll-out Index(VRI) Vs. Population ages 65 and above (% of total population)
Population ages 65 and above (% of total population)
0
5
10
15
20
25
0.00
0.02
0.04
0.06
0.08
Vaccine Roll-out Index(VRI)

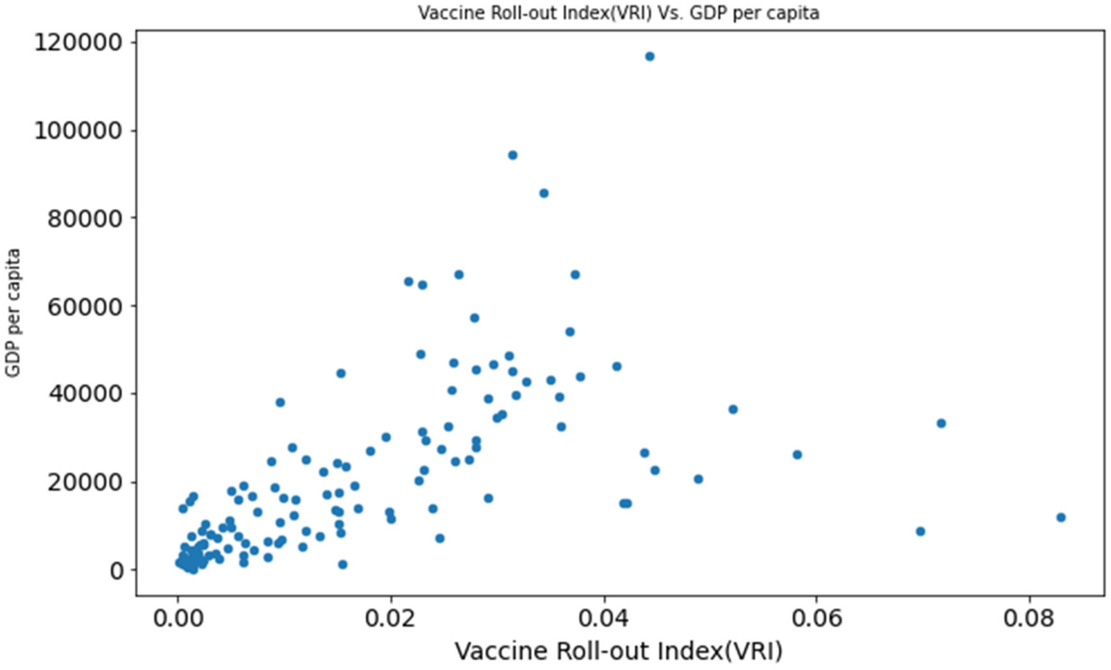
Vaccine Roll-out Index(VRI) Vs. GDP per capita
GDP per capita
0
20000
40000
60000
80000
100000
120000
0.00
0.02
0.04
0.06
0.08
Vaccine Roll-out Index(VRI)

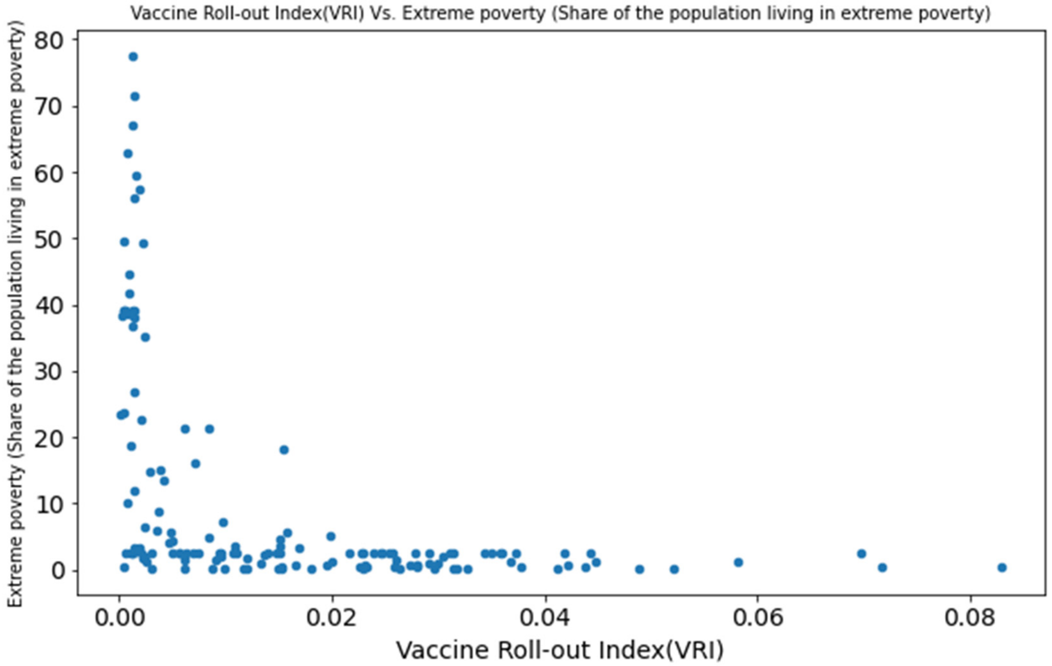
Vaccine Roll-out Index(VRI) Vs. Extreme poverty (Share of the population living in extreme poverty)
Extreme poverty (Share of the population living in extreme poverty)
80
70
60
50
40
30
20
10
0
0.00
0.02
0.04
0.06
0.08
Vaccine Roll-out Index(VRI)

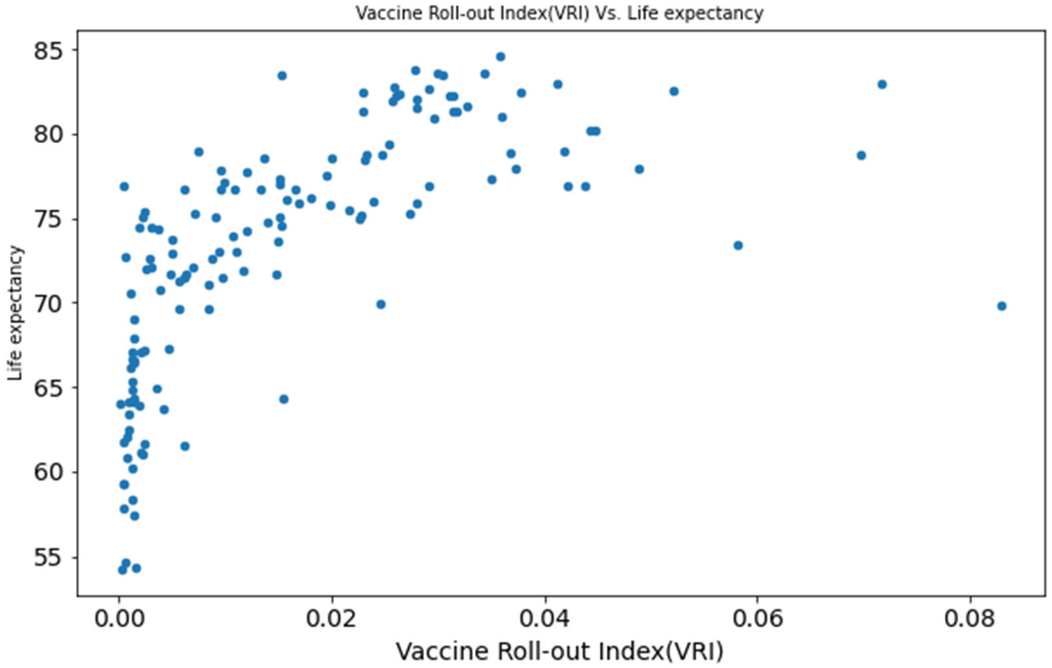
Vaccine Roll-out Index(VRI) Vs. Life expectancy
Life expectancy
85
80
75
70
65
60
55
0.00
0.02
0.04
0.06
0.08
Vaccine Roll-out Index(VRI)

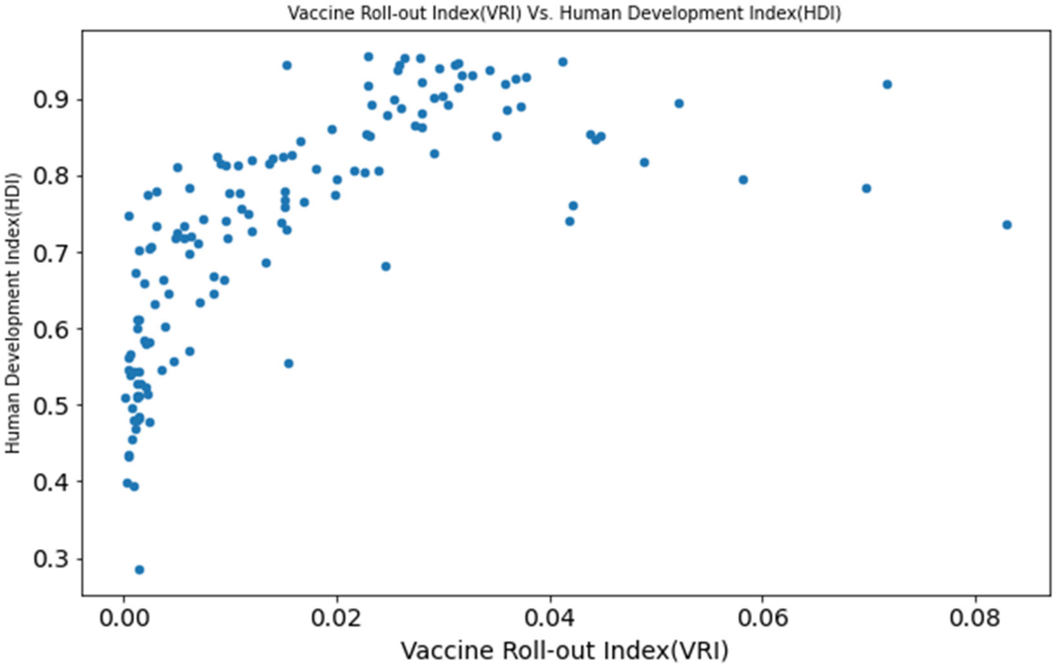
Vaccine Roll-out Index(VRI) Vs. Human Development Index(HDI)
Human Development Index(HDI)
0.9
0.8
0.7
0.6
0.5
0.4
0.3
0.00
0.02
0.04
0.06
0.08
Vaccine Roll-out Index(VRI)

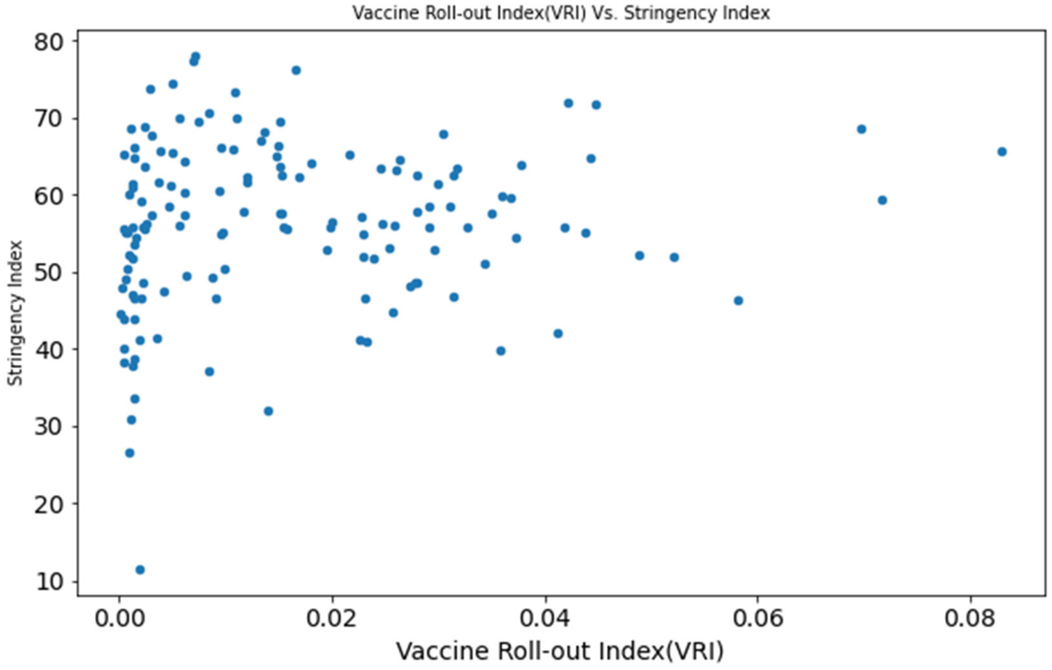
Vaccine Roll-out Index(VRI) Vs. Stringency Index
Stringency Index
80
70
60
50
40
30
20
10
0.00
0.02
0.04
0.06
0.08
Vaccine Roll-out Index(VRI)

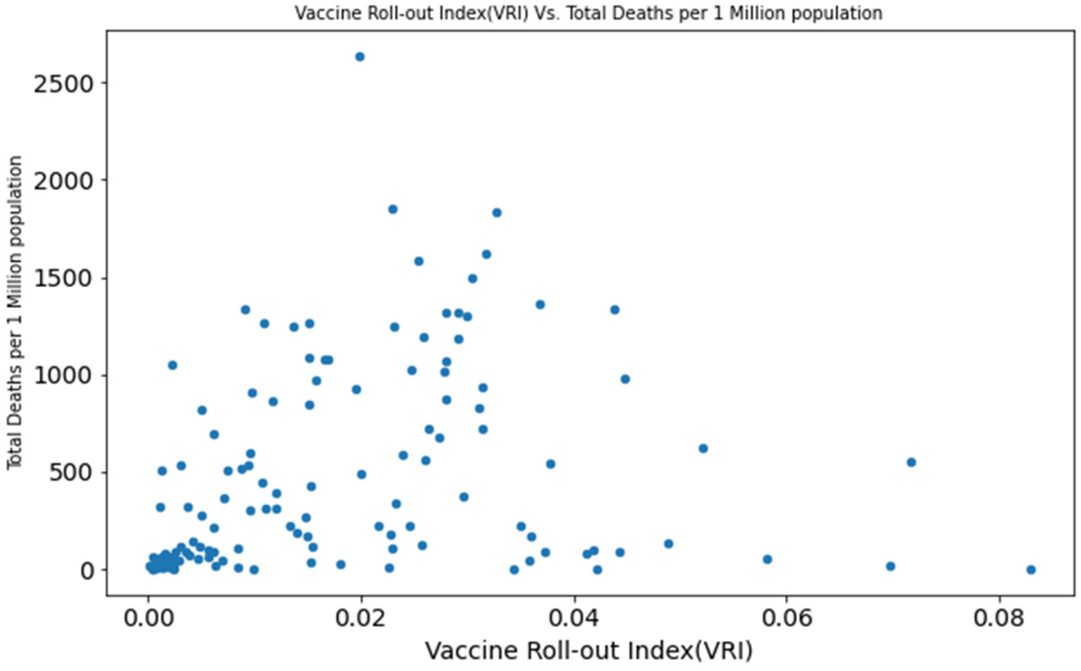
Vaccine Roll-out Index(VRI) Vs. Total Deaths per 1 Million population
Total Deaths per 1 Million population
Vaccine Roll-out Index(VRI)
0
500
1000
1500
2000
2500
0.00
0.02
0.04
0.06
0.08

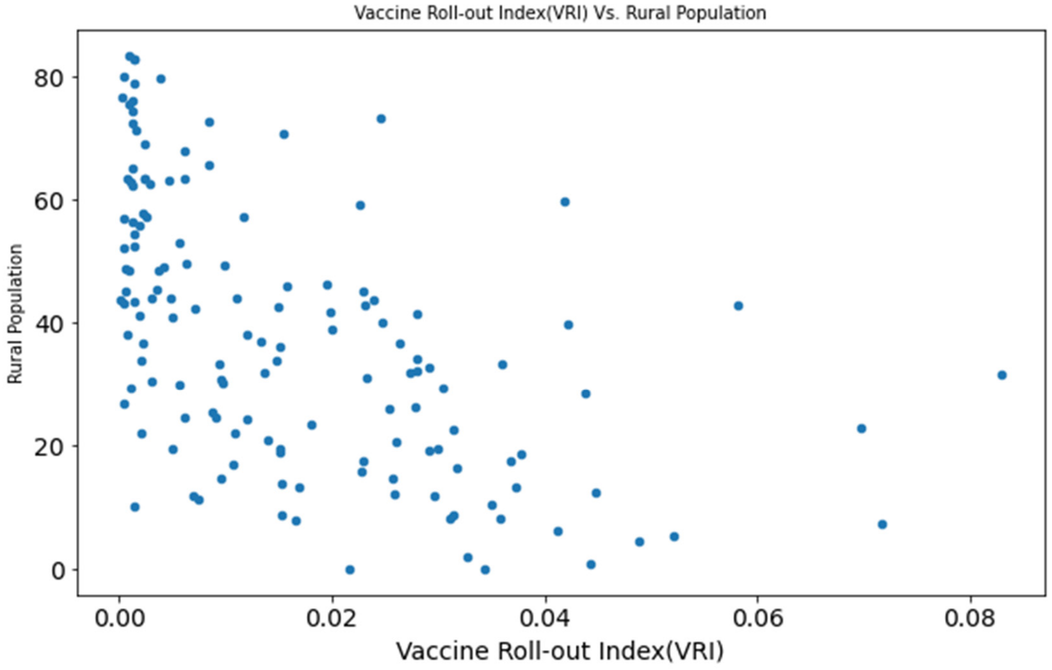
Vaccine Roll-out Index(VRI) Vs. Rural Population
Rural Population
Vaccine Roll-out Index(VRI)
0
20
40
60
80
0.00
0.02
0.04
0.06
0.08

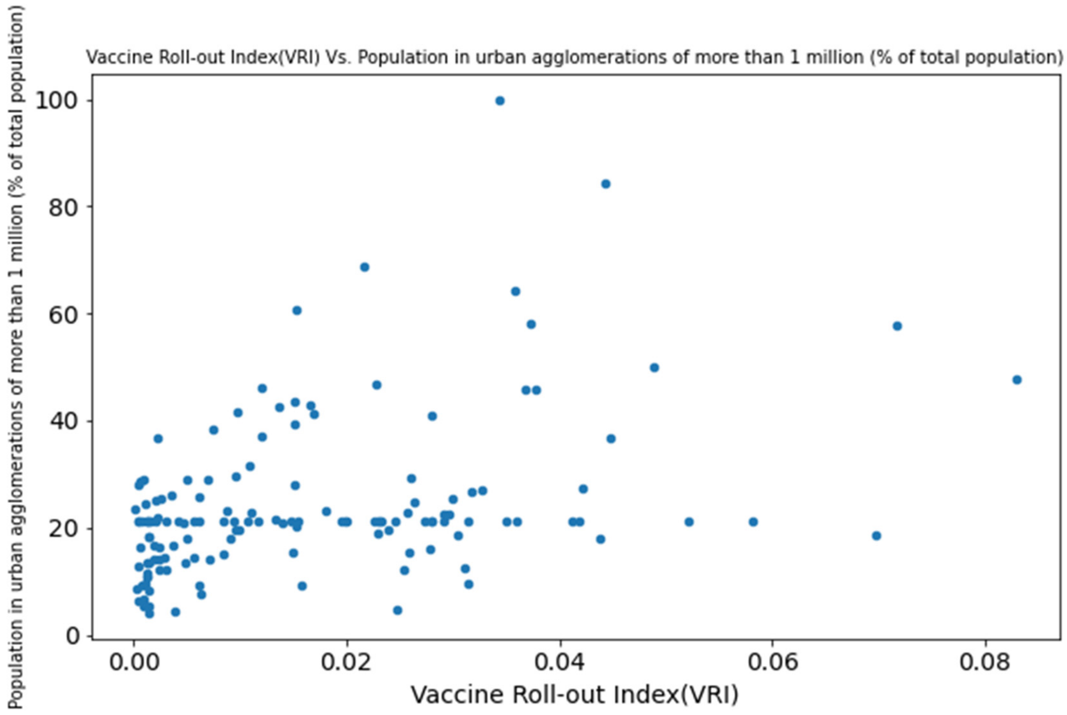
Vaccine Roll-out Index(VRI) Vs. Population in urban agglomerations of more than 1 million (% of total population)
Population in urban agglomerations of more than 1 million (% of total population)
0
20
40
60
80
100
0.00
0.02
0.04
0.06
0.08
Vaccine Roll-out Index(VRI)

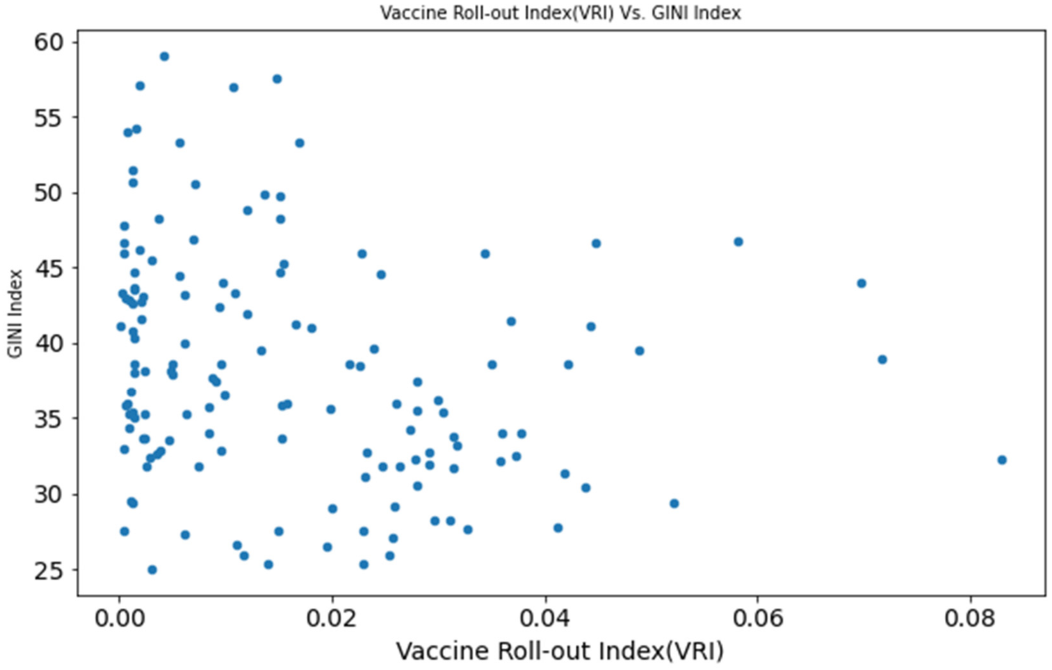
Vaccine Roll-out Index(VRI) Vs. GINI Index
GINI Index
25
30
35
40
45
50
55
60
0.00
0.02
0.04
0.06
0.08
Vaccine Roll-out Index(VRI)

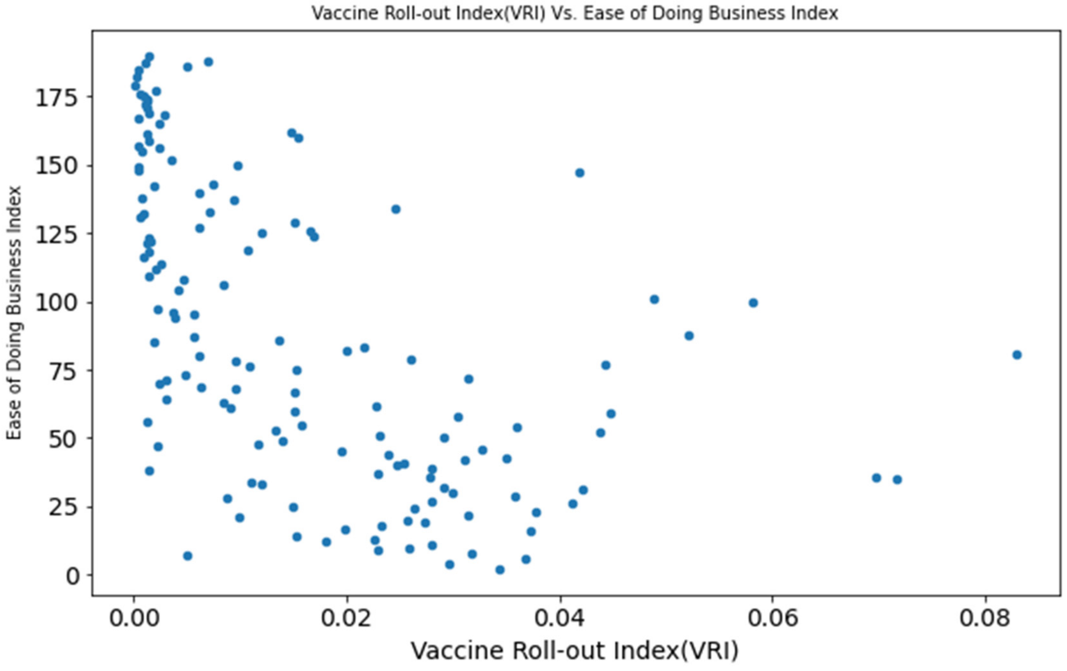

Vaccine Roll-out Index(VRI) Vs. Ease of Doing Business Index
Ease of Doing Business Index
Vaccine Roll-out Index(VRI)
0
25
50
75
100
125
150
175
0.00
0.02
0.04
0.06
0.08

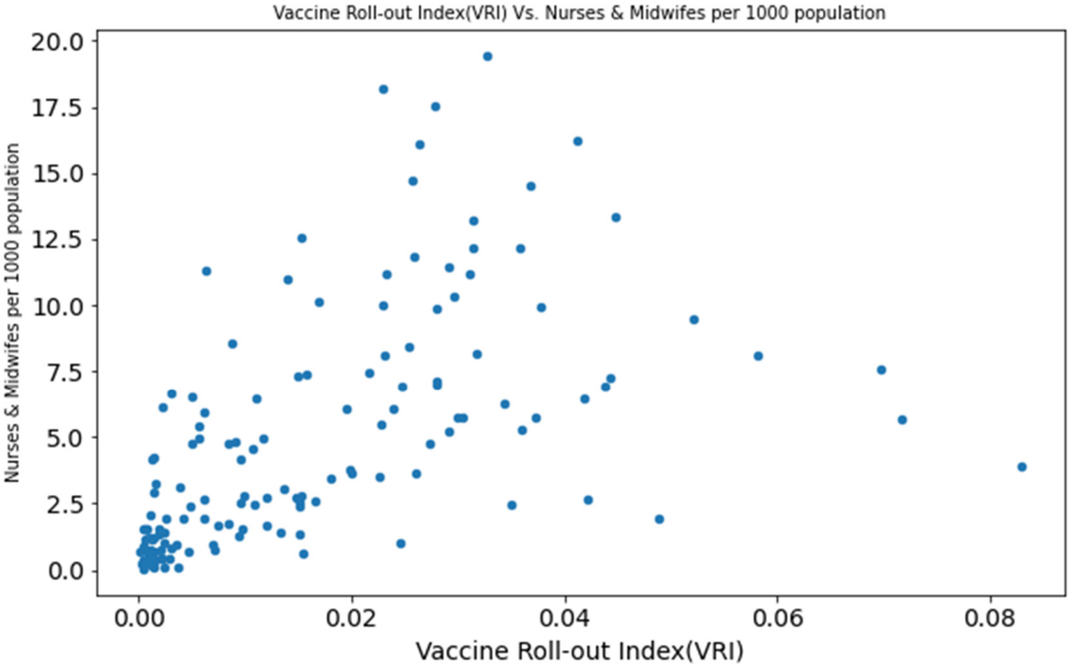

Vaccine Roll-out Index(VRI) Vs. Nurses & Midwifes per 1000 population
Nurses & Midwifes per 1000 population
Vaccine Roll-out Index(VRI)
0.0
2.5
5.0
7.5
10.0
12.5
15.0
17.5
20.0
0.00
0.02
0.04
0.06
0.08

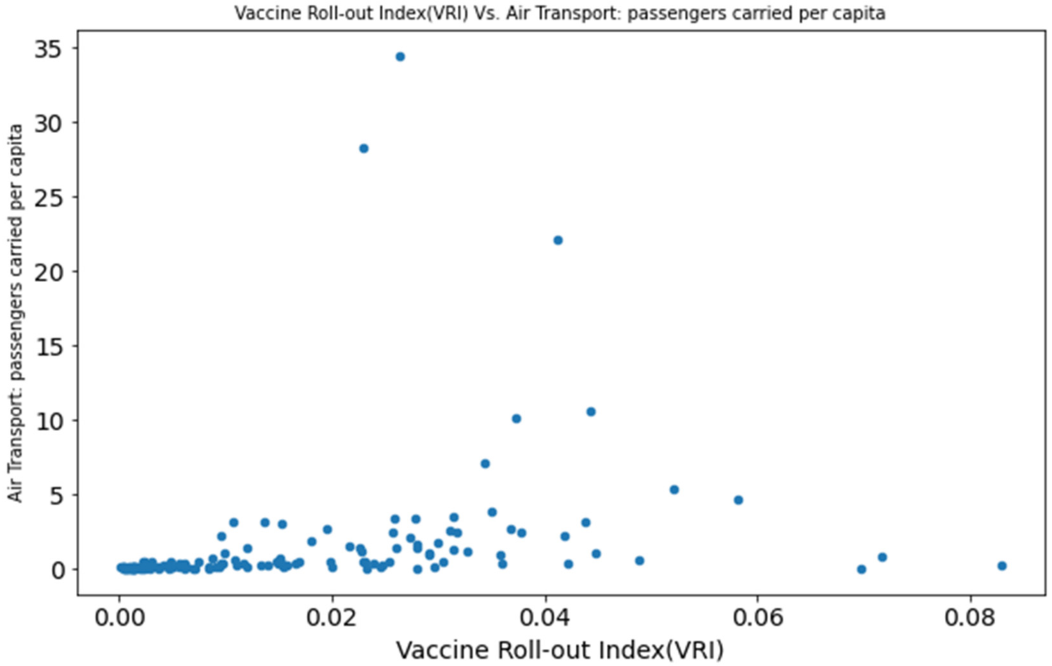
Vaccine Roll-out Index(VRI) Vs. Air Transport: passengers carried per capita
Air Transport: passengers carried per capita
Vaccine Roll-out Index(VRI)
0
5
10
15
20
25
30
35
0.00
0.02
0.04
0.06
0.08

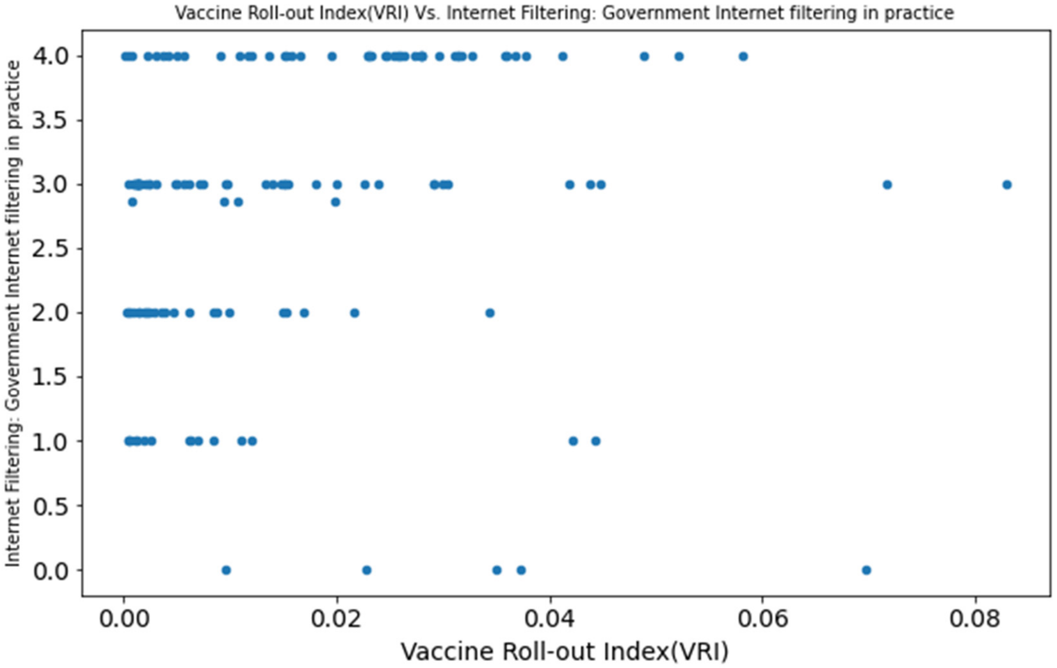
Vaccine Roll-out Index(VRI) Vs. Internet Filtering: Government Internet filtering in practice
Internet Filtering: Government Internet filtering in practice
Vaccine Roll-out Index(VRI)
0.0
0.5
1.0
1.5
2.0
2.5
3.0
3.5
4.0
0.00
0.02
0.04
0.06
0.08

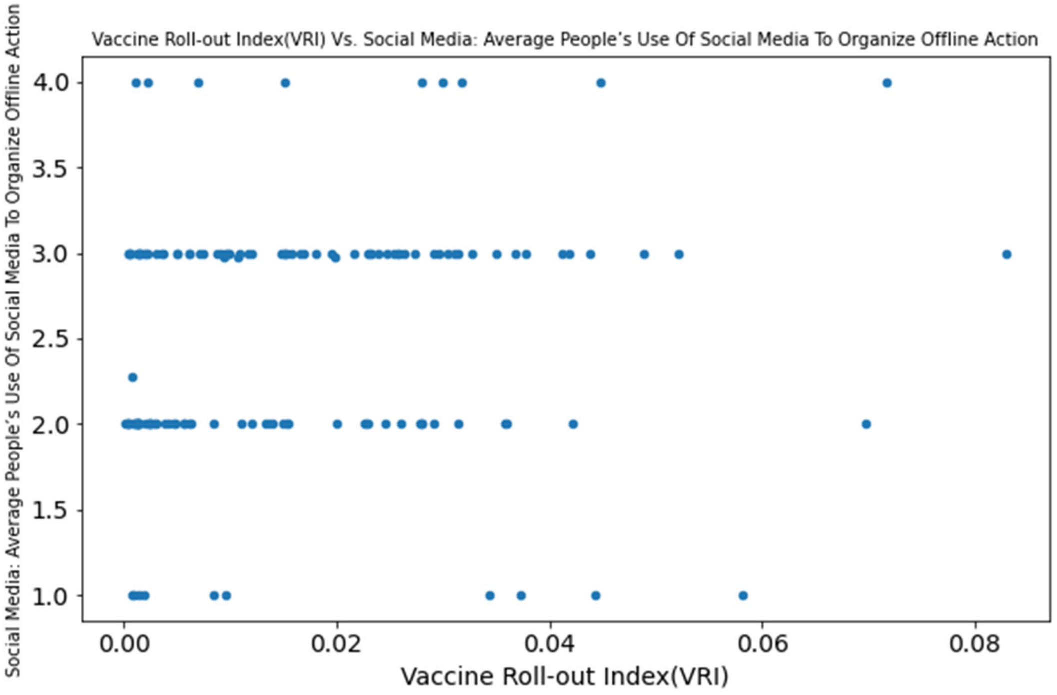
Vaccine Roll-out Index(VRI) Vs. Social Media: Average People's Use Of Social Media To Organize Offline Action
Social Media: Average People's Use Of Social Media To Organize Offline Action
Vaccine Roll-out Index(VRI)
4.0
3.5
3.0
2.5
2.0
1.5
1.0
0.00
0.02
0.04
0.06
0.08

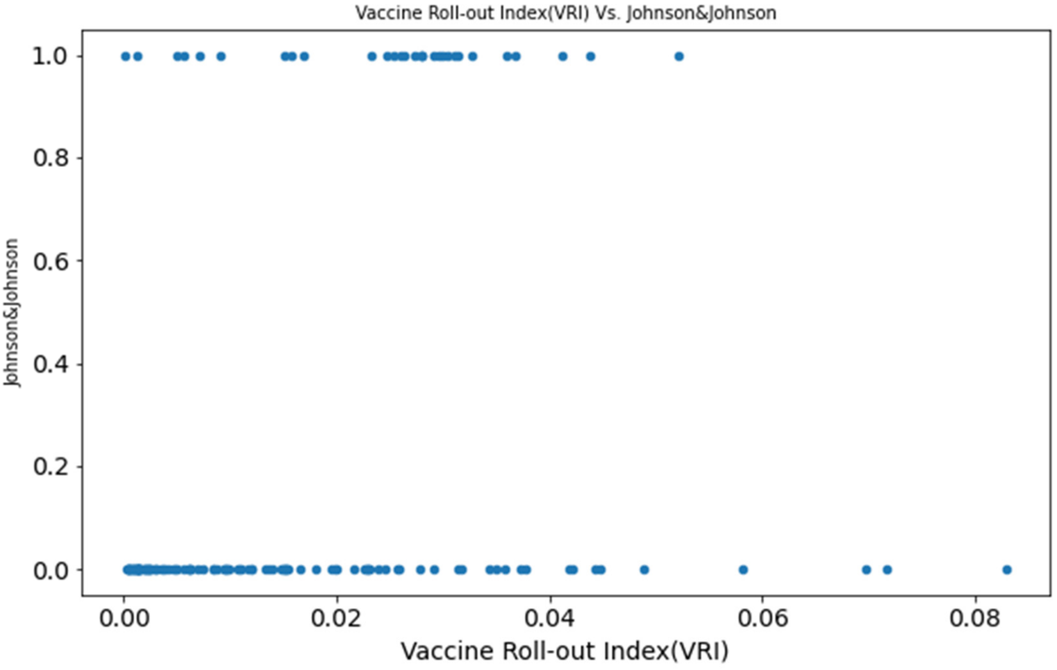
Vaccine Roll-out Index(VRI) Vs. Johnson&Johnson
Johnson&Johnson
Vaccine Roll-out Index(VRI)
1.0
0.8
0.6
0.4
0.2
0.0
0.00
0.02
0.04
0.06
0.08

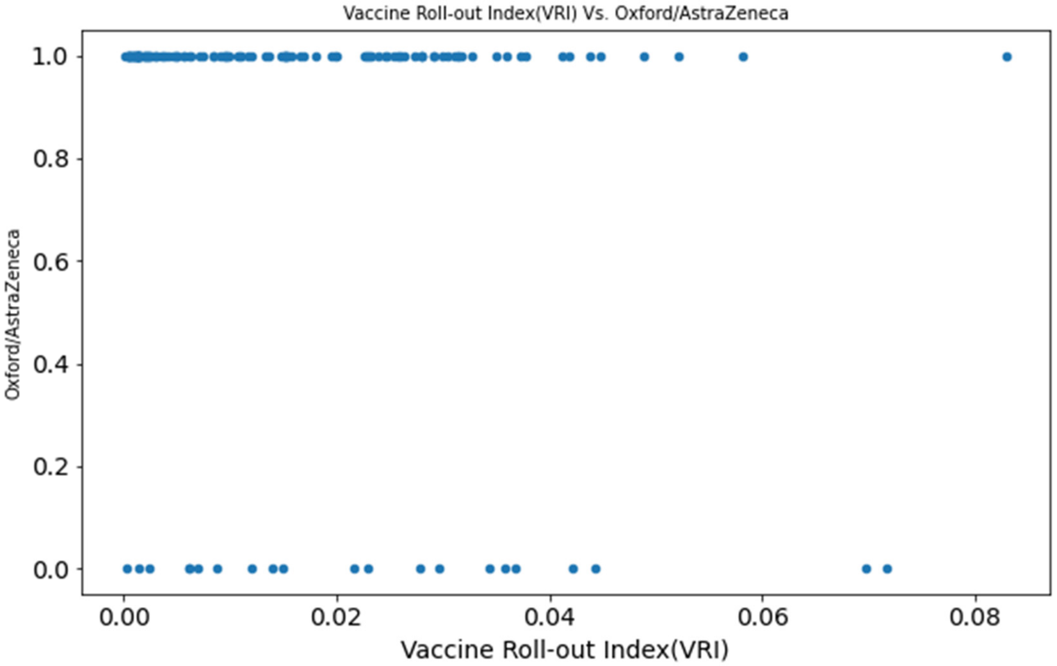

Vaccine Roll-out Index(VRI) Vs. Oxford/AstraZeneca
Oxford/AstraZeneca
1.0
0.8
0.6
0.4
0.2
0.0
0.00
0.02
0.04
0.06
0.08
Vaccine Roll-out Index(VRI)

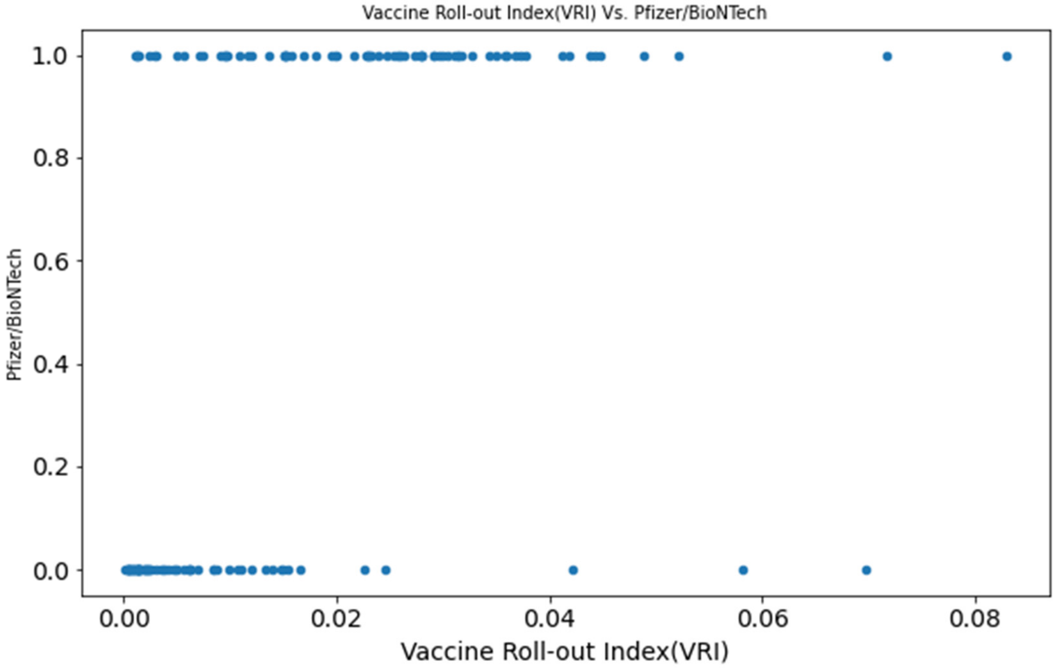

Vaccine Roll-out Index(VRI) Vs. Pfizer/BioNTech
Pfizer/BioNTech
1.0
0.8
0.6
0.4
0.2
0.0
0.00
0.02
0.04
0.06
0.08
Vaccine Roll-out Index(VRI)

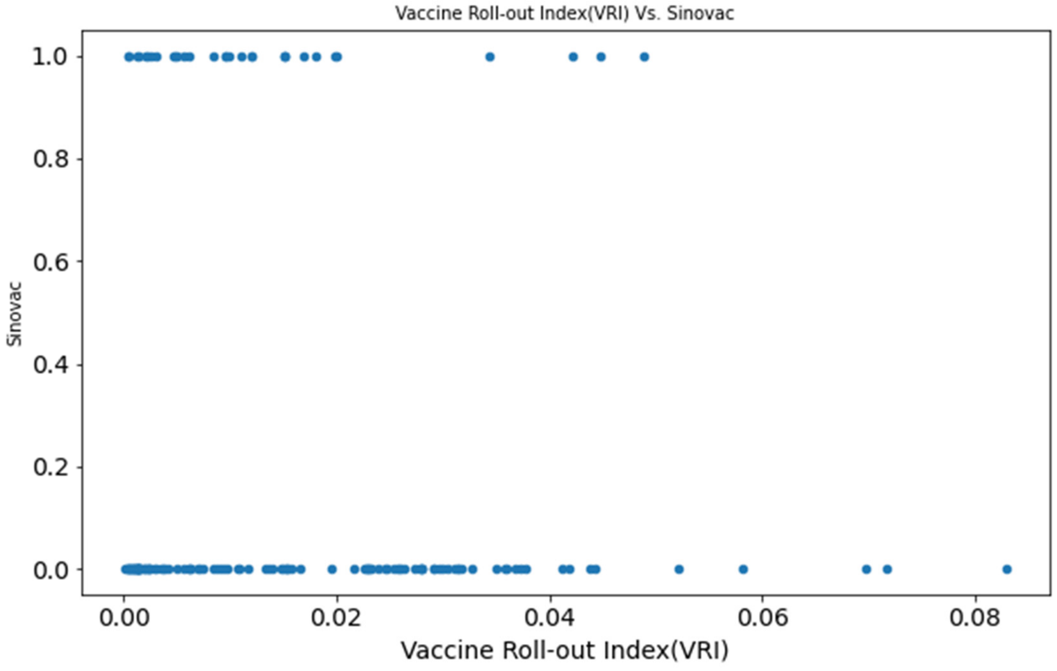
Vaccine Roll-out Index(VRI) Vs. Sinovac
Sinovac
1.0
0.8
0.6
0.4
0.2
0.0
0.00
0.02
0.04
0.06
0.08
Vaccine Roll-out Index(VRI)

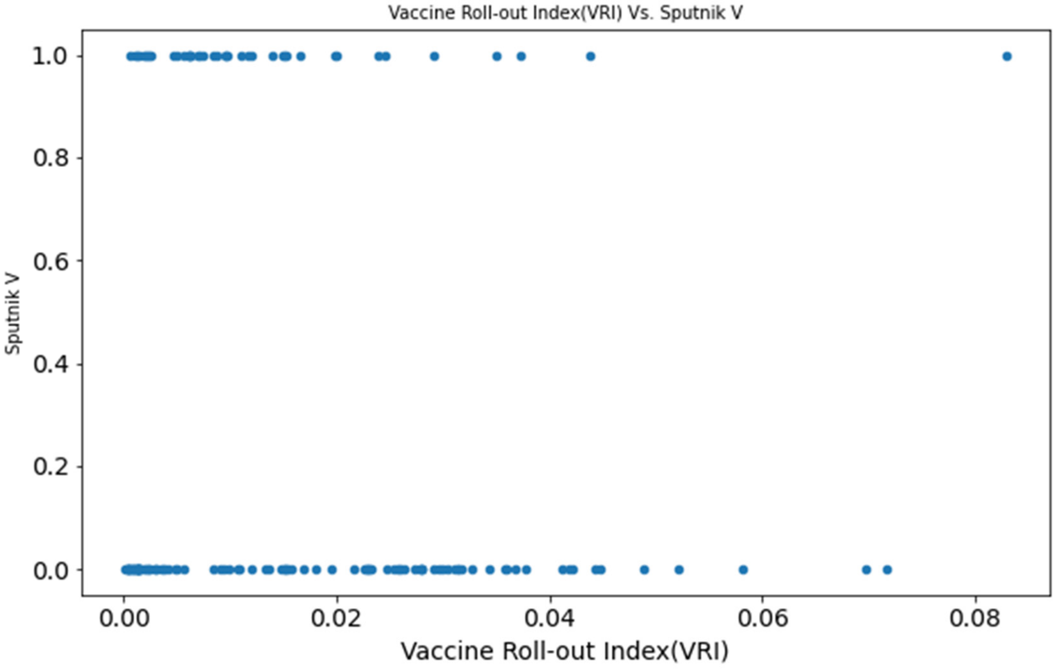
Vaccine Roll-out Index(VRI) Vs. Sputnik V
Sputnik V
1.0
0.8
0.6
0.4
0.2
0.0
0.00
0.02
0.04
0.06
0.08
Vaccine Roll-out Index(VRI)

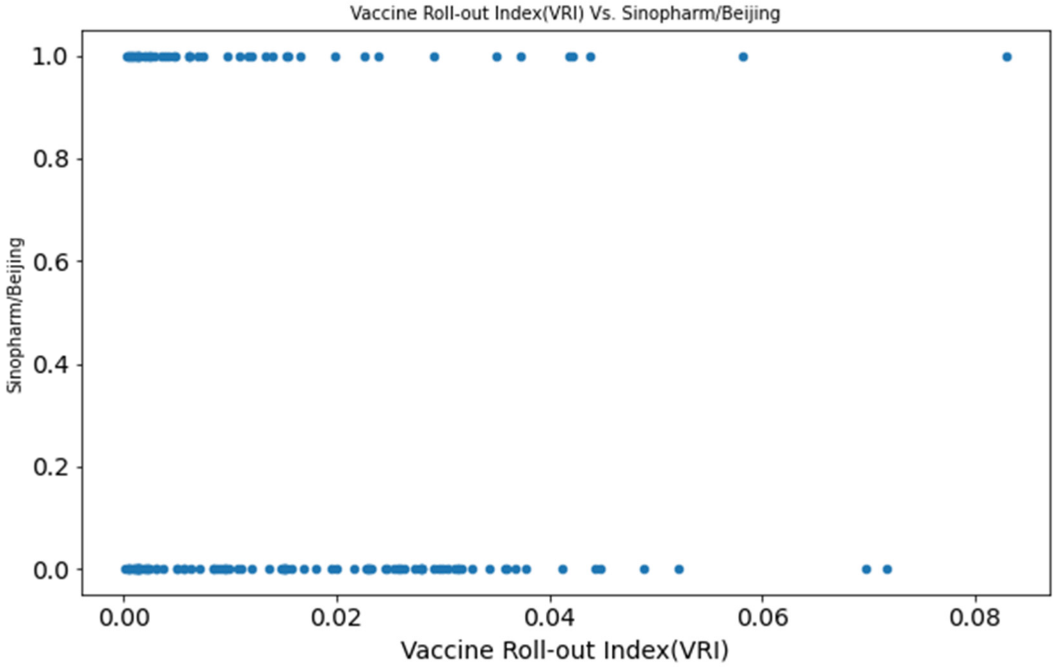

Vaccine Roll-out Index(VRI) Vs. Sinopharm/Beijing
1.0
0.8
0.6
0.4
0.2
0.0
Sinopharm/Beijing
0.00
0.02
0.04
0.06
0.08
Vaccine Roll-out Index(VRI)

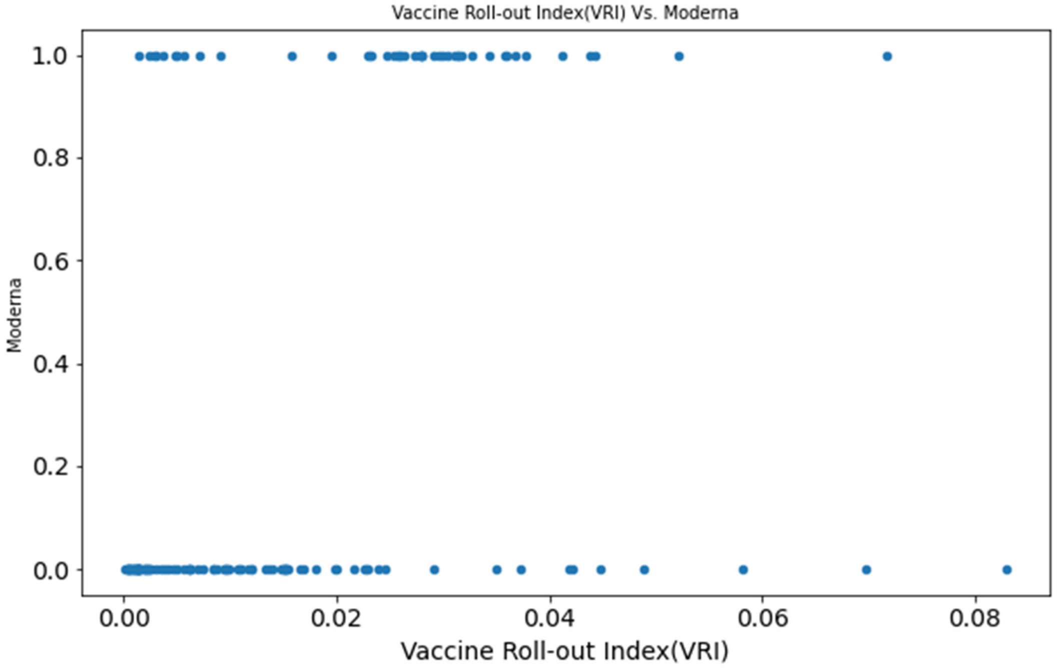

Vaccine Roll-out Index(VRI) Vs. Moderna
1.0
0.8
0.6
0.4
0.2
0.0
Moderna
0.00
0.02
0.04
0.06
0.08
Vaccine Roll-out Index(VRI)

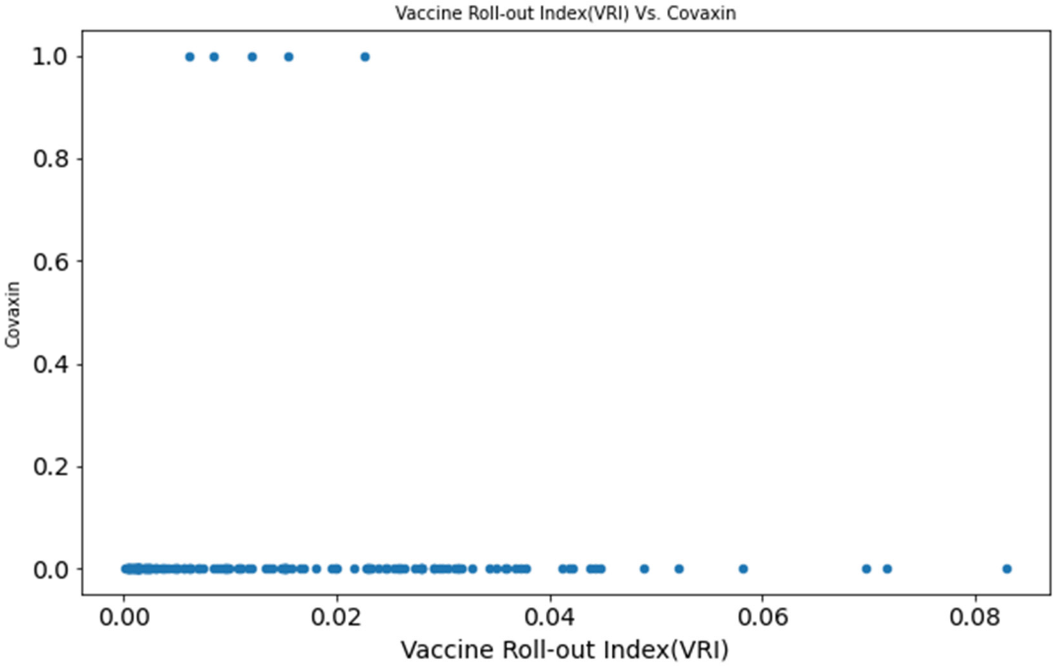
Vaccine Roll-out Index(VRI) Vs. Covaxin
Covaxin
Vaccine Roll-out Index(VRI)
1.0
0.8
0.6
0.4
0.2
0.0
0.00
0.02
0.04
0.06
0.08

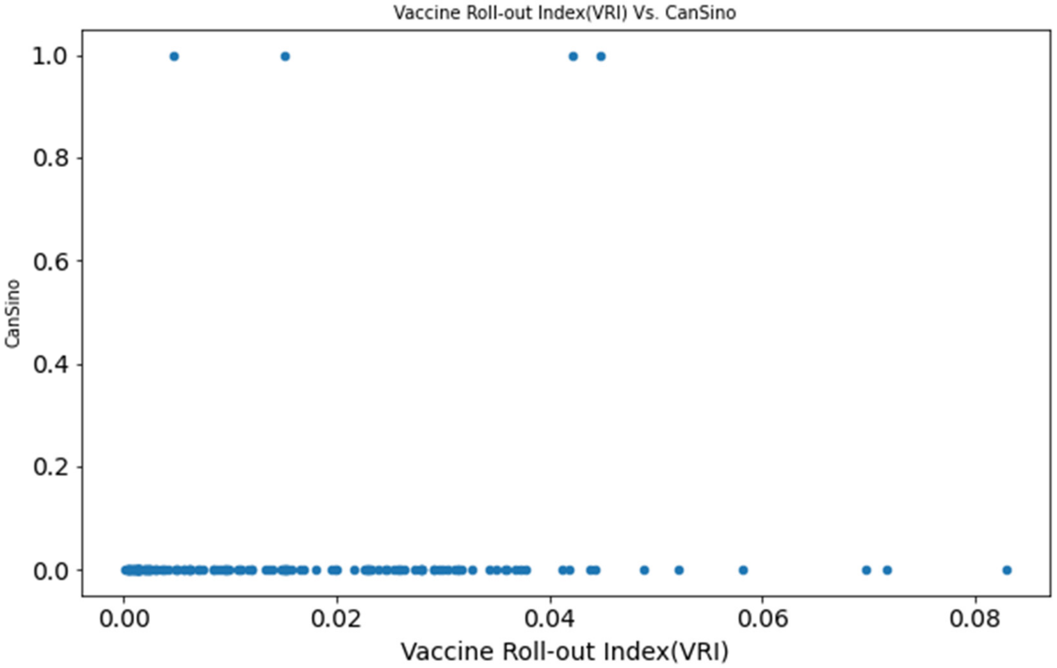
Vaccine Roll-out Index(VRI) Vs. CanSino
CanSino
Vaccine Roll-out Index(VRI)
1.0
0.8
0.6
0.4
0.2
0.0
0.00
0.02
0.04
0.06
0.08

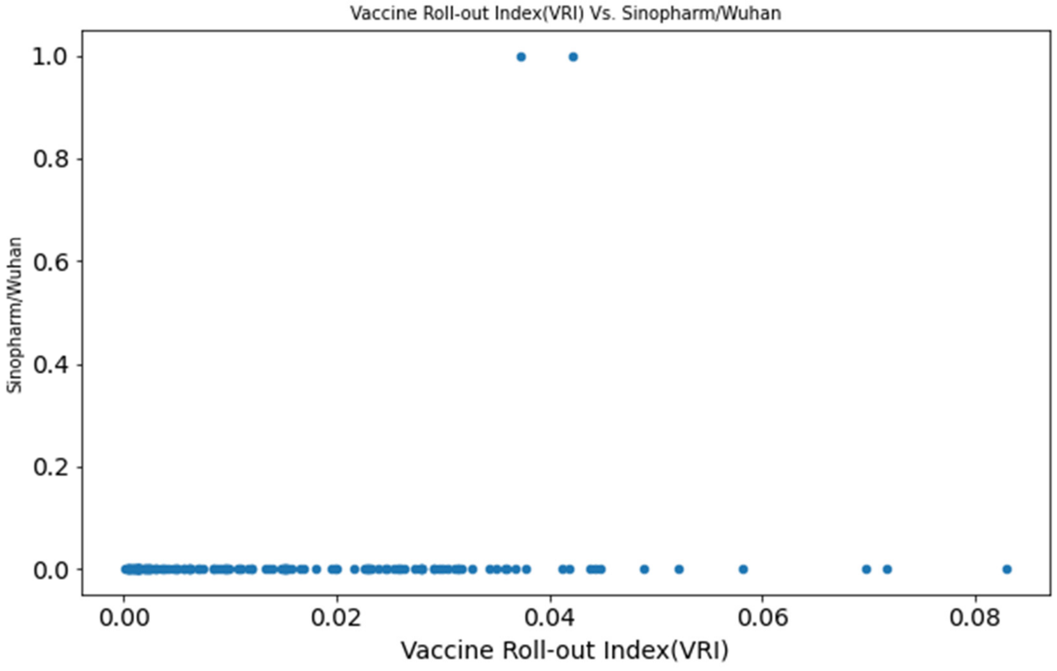
Vaccine Roll-out Index(VRI) Vs. Sinopharm/Wuhan
Sinopharm/Wuhan
Vaccine Roll-out Index(VRI)
1.0
0.8
0.6
0.4
0.2
0.0
0.00
0.02
0.04
0.06
0.08

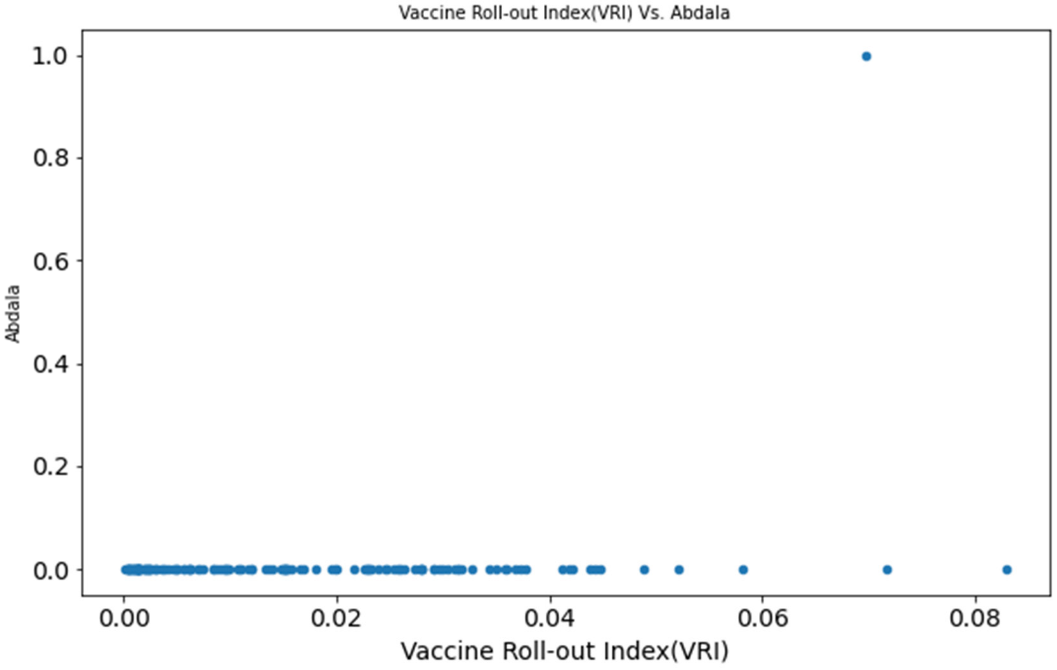
Vaccine Roll-out Index(VRI) Vs. Abdala
Abdala
Vaccine Roll-out Index(VRI)
1.0
0.8
0.6
0.4
0.2
0.0
0.00
0.02
0.04
0.06
0.08

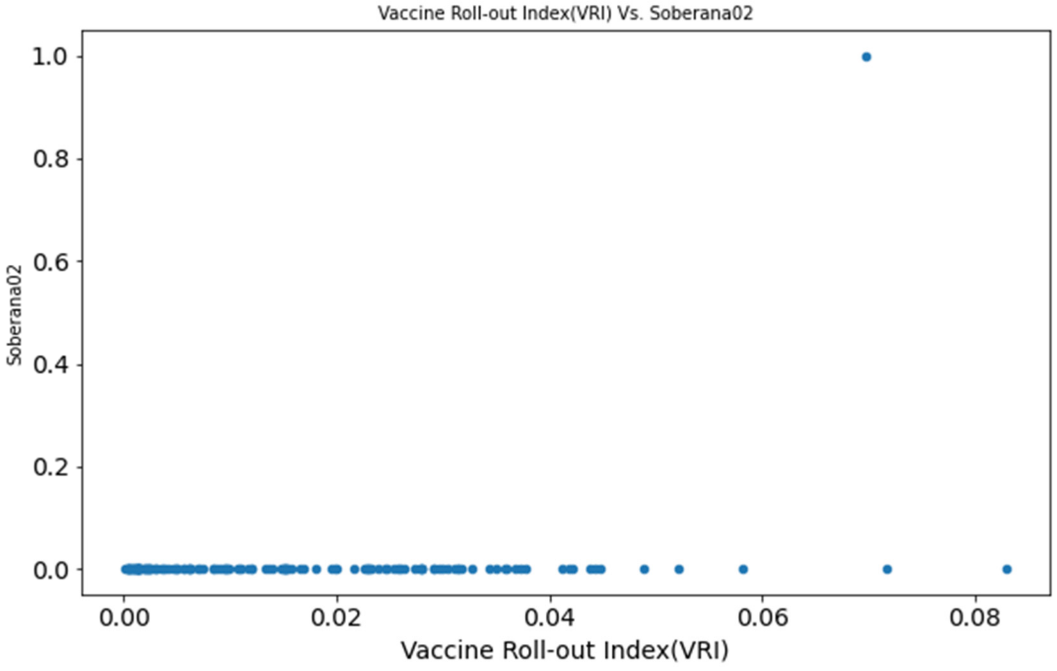

Vaccine Roll-out Index(VRI) Vs. Soberana02
1.0
0.8
0.6
0.4
0.2
0.0
Soberana02
0.00
0.02
0.04
0.06
0.08
Vaccine Roll-out Index(VRI)

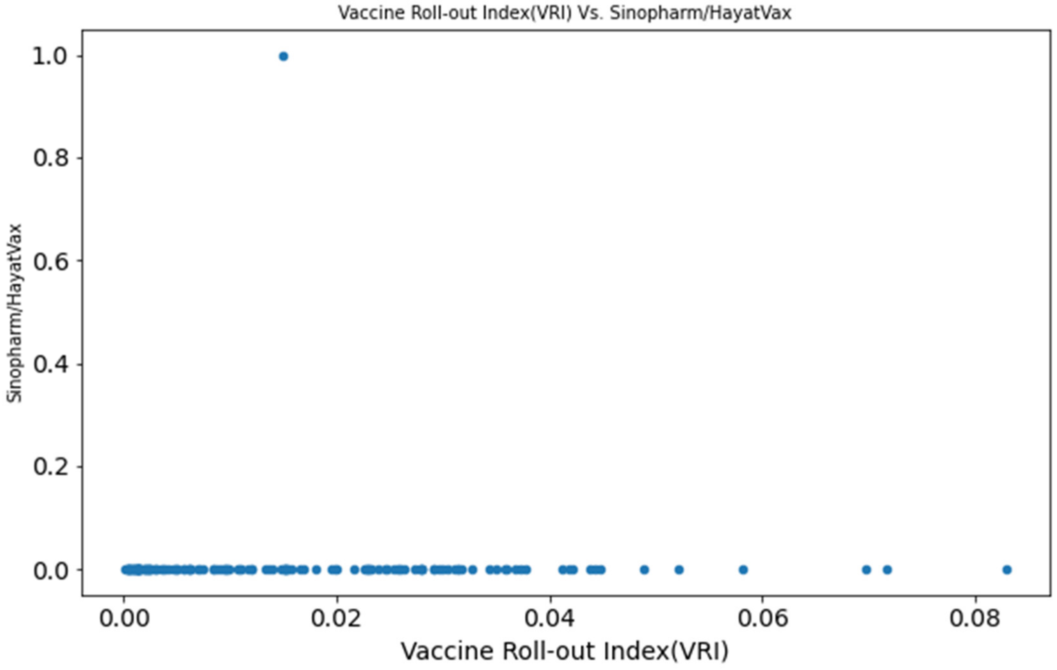

Vaccine Roll-out Index(VRI) Vs. Sinopharm/HayatVax
1.0
0.8
0.6
0.4
0.2
0.0
Sinopharm/HayatVax
0.00
0.02
0.04
0.06
0.08
Vaccine Roll-out Index(VRI)

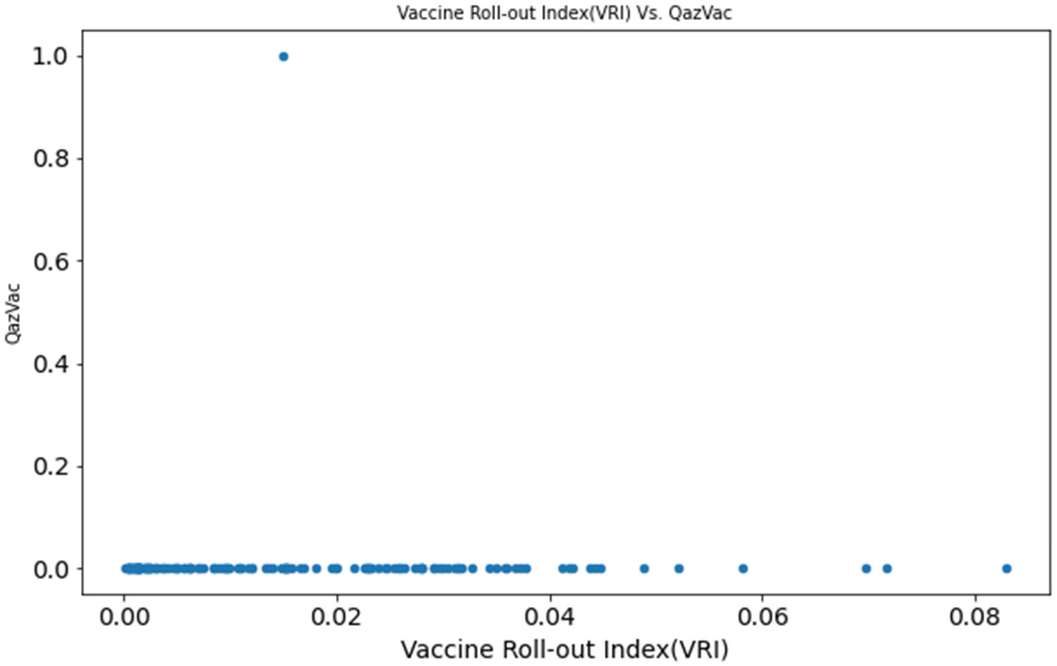

Vaccine Roll-out Index(VRI) Vs. QazVac
QazVac
1.0
0.8
0.6
0.4
0.2
0.0
0.00
0.02
0.04
0.06
0.08
Vaccine Roll-out Index(VRI)

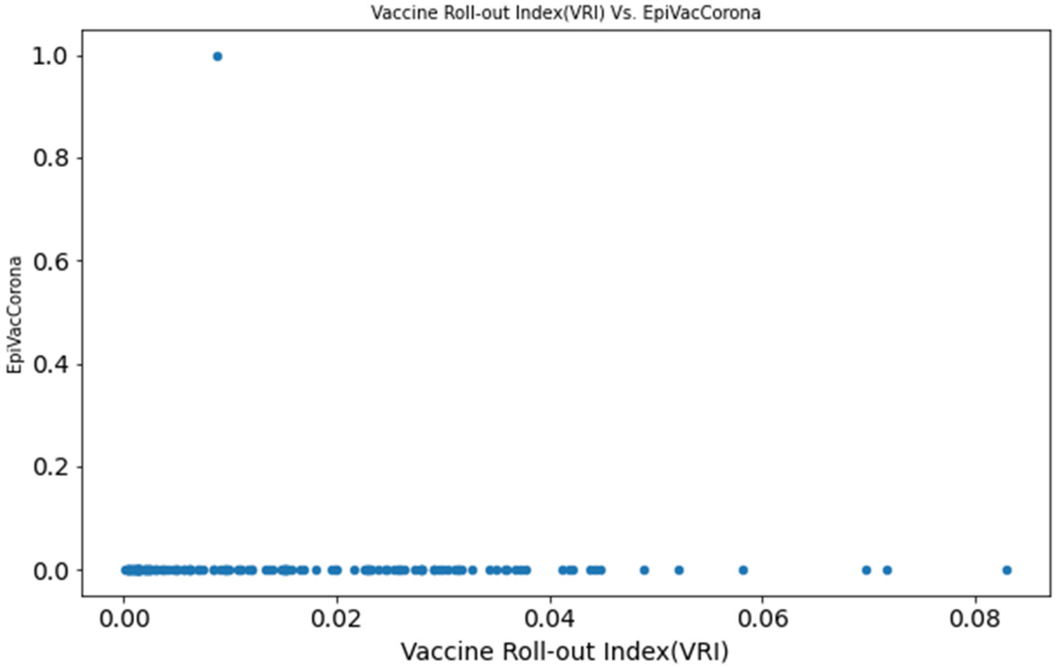

Vaccine Roll-out Index(VRI) Vs. EpiVacCorona
EpiVacCorona
1.0
0.8
0.6
0.4
0.2
0.0
0.00
0.02
0.04
0.06
0.08
Vaccine Roll-out Index(VRI)

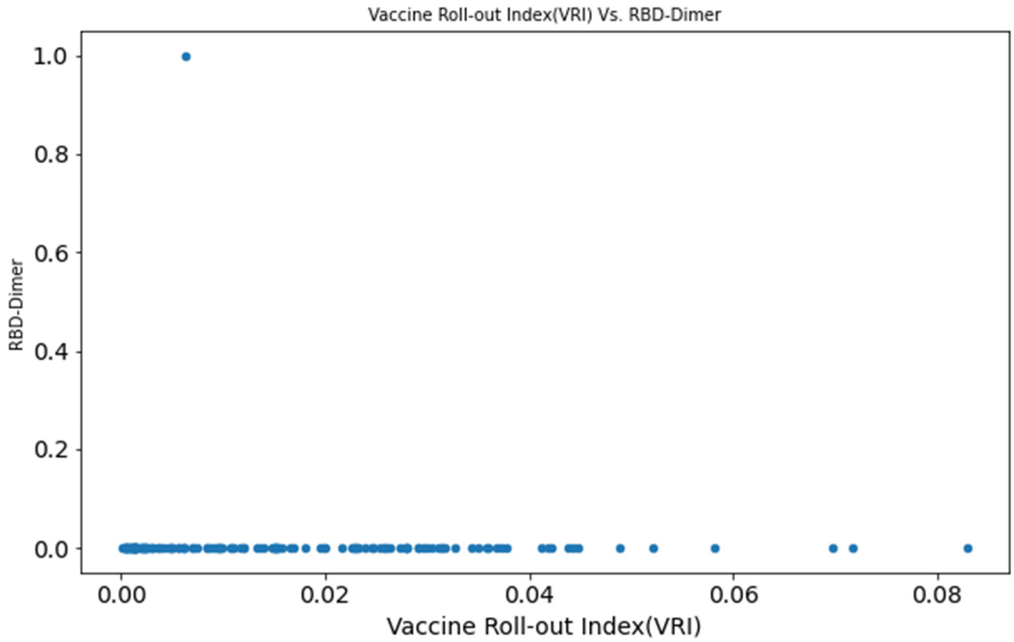

Table S1. Estimated vaccination uptake rates across studied countries.

Country	Vaccination uptake rate(r)
Afghanistan	0.036886
Albania	0.050539
Algeria	0.238886
Angola	0.041562
Argentina	0.024316
Armenia	0.043949
Australia	0.033448
Austria	0.026451
Azerbaijan	0.023641
Bahamas	0.040445
Bahrain	0.02601
Bangladesh	0.04429
Belarus	0.059885
Belgium	0.027123
Benin	0.098466
Bolivia	0.036471
Botswana	0.040315
Brazil	0.026061
Bulgaria	0.031243
Cameroon	0.02808
Canada	0.029401
Cape Verde	0.032556
Chad	0.120965
Chile	0.033766
China	0.037979
Colombia	0.029999
Comoros	0.105911
Croatia	0.03123
Cuba	0.088333

Cyprus	0.032356
Czechia	0.026624
Denmark	0.023935
Djibouti	0.07632
Ecuador	0.026507
Egypt	0.04985
Estonia	0.027471
Eswatini	0.02077
Ethiopia	0.068304
Finland	0.026239
France	0.027228
Gabon	0.036543
Gambia	0.040028
Georgia	0.045149
Germany	0.028984
Ghana	0.03259
Greece	0.026333
Guatemala	0.03564
Guinea	0.038233
Guinea-Bissau	0.098046
Guyana	0.050397
Haiti	0.39486
Honduras	0.040873
Hungary	0.041707
Iceland	0.030035
India	0.025807
Indonesia	0.020553
Iran	0.050244
Iraq	0.03765
Ireland	0.022765
Israel	0.05577
Italy	0.027421
Jamaica	0.029102
Japan	0.054732
Jordan	0.030972
Kazakhstan	0.03233
Kenya	0.041444
Kuwait	0.038796
Kyrgyzstan	0.077308
Latvia	0.038632
Lebanon	0.026569
Lesotho	0.043525
Liberia	0.049799
Libya	0.062362
Lithuania	0.02914
Luxembourg	0.028399
Madagascar	0.172796
Malawi	0.056374
Malaysia	0.030907
Maldives	0.038866
Mali	0.050372
Malta	0.030672

Mauritania	0.083366
Mauritius	0.024815
Mexico	0.030706
Moldova	0.046353
Mongolia	0.065861
Montenegro	0.056839
Morocco	0.021285
Mozambique	0.035554
Myanmar	0.037374
Namibia	0.053373
Nepal	0.02227
Netherlands	0.026902
Nicaragua	0.030295
Niger	0.047662
Nigeria	0.028309
North Macedonia	0.048717
Norway	0.023933
Oman	0.025975
Pakistan	0.038974
Panama	0.026058
Paraguay	0.040498
Peru	0.028688
Philippines	0.035085
Poland	0.027506
Portugal	0.024118
Qatar	0.034198
Romania	0.032268
Russia	0.021375
Rwanda	0.027229
Saudi Arabia	0.0306
Senegal	0.025564
Serbia	0.029498
Seychelles	0.040501
Singapore	0.027547
Slovakia	0.025418
Slovenia	0.027816
Somalia	0.091266
South Sudan	0.066069
Spain	0.025311
Sudan	0.070128
Suriname	0.035867
Sweden	0.025062
Switzerland	0.027085
Syria	0.085745
Tajikistan	0.162215
Thailand	0.043262
Togo	0.041384
Tunisia	0.04269
Turkey	0.014432
Uganda	0.038506
Ukraine	0.025742
United Arab Emirates	0.02213

United Kingdom	0.025509
Uruguay	0.035843
USA	0.035458
Uzbekistan	0.0365
Venezuela	0.049466
Vietnam	0.046402
Yemen	0.109131
Zambia	0.083315
Zimbabwe	0.041847

Table S2. Vaccine Roll-Out Index (VRI) values for the countries studied in the present investigation.

Country	Vaccine Roll-out Index(VRI)
Afghanistan	0.001210084
Albania	0.019998731
Algeria	0.000376271
Angola	0.002013889
Argentina	0.016492992
Armenia	0.002262223
Australia	0.015207455
Austria	0.028058527
Azerbaijan	0.010991486
Bahamas	0.010610271
Bahrain	0.034968284
Bangladesh	0.002936957
Belarus	0.013910751
Belgium	0.032756669
Benin	0.000470157
Bolivia	0.009740326
Botswana	0.005681481
Brazil	0.01690497
Bulgaria	0.009000794
Cameroon	0.000351055
Canada	0.037804863
Cape Verde	0.009336396
Chad	0.000212166
Chile	0.044754816
China	0.042251672
Colombia	0.015117204
Comoros	0.015341107
Croatia	0.023062005
Cuba	0.069760121
Cyprus	0.036013351
Czech Republic	0.025339535
Denmark	0.029578202
Djibouti	0.002069905
Ecuador	0.015100925
Egypt	0.002600051
Estonia	0.023204433
Eswatini	0.001175613
Ethiopia	0.0013058
Finland	0.025772769

France	0.029070493
Gabon	0.001431377
Gambia	0.000721449
Georgia	0.004898321
Germany	0.031479699
Ghana	0.001333465
Greece	0.026033766
Guatemala	0.003734472
Guinea	0.002328672
Guinea-Bissau	0.001256714
Guyana	0.024537109
Haiti	0.000141218
Honduras	0.007040066
Hungary	0.043844583
Iceland	0.041227471
India	0.008428507
Indonesia	0.004872036
Iran	0.00608283
Iraq	0.00101829
Ireland	0.026399925
Israel	0.07166115
Italy	0.030387715
Jamaica	0.002957235
Japan	0.035741875
Jordan	0.015264555
Kazakhstan	0.014923595
Kenya	0.001297087
Kuwait	0.02157986
Kyrgyzstan	0.006139407
Latvia	0.027341645
Lebanon	0.007399561
Lesotho	0.001482114
Liberia	0.000939556
Libya	0.004962114
Lithuania	0.027962536
Luxembourg	0.031362136
Madagascar	0.001229315
Malawi	0.001321412
Malaysia	0.018091996
Maldives	0.041921256
Mali	0.000489675
Malta	0.052144116
Mauritania	0.003444146
Mauritius	0.022565533
Mexico	0.015078367
Moldova	0.011592435
Mongolia	0.082917821
Montenegro	0.029209376
Morocco	0.013255739
Mozambique	0.000786079
Myanmar	0.002404144
Namibia	0.004215661

Nepal	0.003857821
Netherlands	0.03101026
Nicaragua	0.001900779
Niger	0.000833531
Nigeria	0.000540932
North Macedonia	0.019813797
Norway	0.02288207
Oman	0.009600429
Pakistan	0.00463121
Panama	0.013600718
Paraguay	0.012059904
Peru	0.010894898
Philippines	0.005607966
Poland	0.024780549
Portugal	0.028033677
Qatar	0.044285025
Romania	0.015680686
Russia	0.008674269
Rwanda	0.001359975
Saudi Arabia	0.022741332
Senegal	0.001384097
Serbia	0.023865134
Seychelles	0.058249532
Singapore	0.034397829
Slovakia	0.019527491
Slovenia	0.022855304
Somalia	0.001434406
South Sudan	0.000336367
Spain	0.029874942
Sudan	0.001296326
Suriname	0.014761417
Sweden	0.025806435
Switzerland	0.027777368
Syria	0.000642921
Tajikistan	0.008368996
Thailand	0.009892484
Togo	0.002270509
Tunisia	0.009620598
Turkey	0.012053051
Uganda	0.000955504
Ukraine	0.003075742
United Arab Emirates	0.03724404
United Kingdom	0.031676818
United States	0.03678192
Uruguay	0.048938744
Uzbekistan	0.006328768
Venezuela	0.006958236
Vietnam	0.002389816
Yemen	0.001139693
Zambia	0.001815672
Zimbabwe	0.006134218
